# EEG datasets for motor imagery brain–computer interface

**DOI:** 10.1093/gigascience/gix034

**Published:** 2017-05-04

**Authors:** Hohyun Cho, Minkyu Ahn, Sangtae Ahn, Moonyoung Kwon, Sung Chan Jun

**Affiliations:** 1 School of Electrical Engineering and Computer Science, Gwangju Institute of Science and Technology, 123 Cheomdangwagi-ro, Buk-gu, Gwangju 61005, Korea; 2 School of Computer Science and Electrical Engineering, Handong Global University, 558 Handong-ro Buk-gu, Pohang Gyeongbuk 37554, Korea; 3 Department of Psychiatry, School of Medicine, University of North Carolina at Chapel Hill, 115 Mason Farm Road, Chapel Hill, NC 27514, USA

**Keywords:** motor imagery, EEG, brain–computer interface, performance variation, subject-to-subject transfer

## Abstract

**Background:**

Most investigators of brain–computer interface (BCI) research believe that BCI can be achieved through induced neuronal activity from the cortex, but not by evoked neuronal activity. Motor imagery (MI)–based BCI is one of the standard concepts of BCI, in that the user can generate induced activity by imagining motor movements. However, variations in performance over sessions and subjects are too severe to overcome easily; therefore, a basic understanding and investigation of BCI performance variation is necessary to find critical evidence of performance variation. Here we present not only EEG datasets for MI BCI from 52 subjects, but also the results of a psychological and physiological questionnaire, EMG datasets, the locations of 3D EEG electrodes, and EEGs for non-task-related states.

**Findings:**

We validated our EEG datasets by using the percentage of bad trials, event-related desynchronization/synchronization (ERD/ERS) analysis, and classification analysis. After conventional rejection of bad trials, we showed contralateral ERD and ipsilateral ERS in the somatosensory area, which are well-known patterns of MI. Finally, we showed that 73.08% of datasets (38 subjects) included reasonably discriminative information.

**Conclusions:**

Our EEG datasets included the information necessary to determine statistical significance; they consisted of well-discriminated datasets (38 subjects) and less-discriminative datasets. These may provide researchers with opportunities to investigate human factors related to MI BCI performance variation, and may also achieve subject-to-subject transfer by using metadata, including a questionnaire, EEG coordinates, and EEGs for non-task-related states.

## Data Description

### Background and purpose

Motor imagery (MI)–based brain–computer interface (BCI) has attracted great interest recently. Compared with other BCI paradigms, MI BCI can provide users with direct communication without any limb movement or external stimulus (for example, P300-based BCI). MI BCI uses “induced” brain activity [1] from the cortex, rather than “evoked” brain activity. Although the concept of MI BCI is fascinating, it has many obstacles. Among these is the fact that BCI researchers have tended to focus on subject-to-subject transfer (training subject-independent algorithm). To achieve effective subject-to-subject transfer, it is important to understand the variations in performance between subjects [2]. Predicting a subject's performance by using the resting state or the background noise from EEG are some examples of this [3–5].

**Figure 1: fig1:**
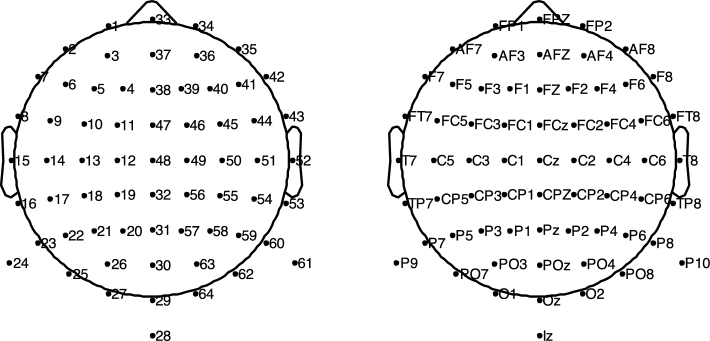
EEG channel configuration—numbering (left) and corresponding labeling (right).

In this[Fig fig1] paper, we recorded MI BCI EEG and EMG datasets simultaneously with 2 classes (100 or 120 trials for each class) from 52 healthy subjects. We also simultaneously collected 20 trials of real hand movement datasets of EEG and EMG for each subject. To study various forms of evidence of performance variation and subject-to-subject transfer, we collected subjective answers to a psychological and physiological questionnaire, as well as EEG results. In addition, we recorded the locations of 3D EEG electrodes and non-task-related EEG (resting state, eyeball and head movements, and jaw clenching). Here, we validated our datasets using the percentage of bad trials, spectral analysis, and classification analysis. These datasets were stored in the *GigaScience* database, GigaDB [6].

### Experimental design

#### Subjects

We conducted a BCI experiment for motor imagery movement (MI movement) of the left and right hands with 52 subjects (19 females, mean age ± SD age = 24.8 ± 3.86 years); the experiment was approved by the Institutional Review Board of Gwangju Institute of Science and Technology. Each subject took part in the same experiment, and subject ID was denoted and indexed as s1, s2, …, s52. Subjects s20 and s33 were both-handed, and the other 50 subjects were right-handed. All subjects gave written informed consent to collect information on brain signals and were paid for their participation. The data collected were used only for research purposes.

#### Recording software and device

EEG data were collected using 64 Ag/AgCl active electrodes. As shown in Fig. 1, a 64-channel montage based on the international 10-10 system was used to record the EEG signals with 512 Hz sampling rates. The EEG device used in this experiment was the Biosemi ActiveTwo system. The BCI2000 system 3.0.2 [7] was used to collect EEG data and present instructions (left hand or right hand MI). Furthermore, we recorded EMG as well as EEG simultaneously with the same system and sampling rate to check actual hand movements. Two EMG electrodes were attached to the flexor digitorum profundus and extensor digitorum on each arm.

For each subject, EEG channel locations (3D coordinates) were collected with a 3D coordinate digitizer (Polhemus Fastrak). Electrode location was measured as the average of three measurements of the digitizer to obtain a stabilized position and prevent hand shaking.

#### Environment

All experiments were conducted at our laboratory during one of four time slots: T1 (9:30–12:00), T2 (12:30–15:00), T3 (15:30–18:00), or T4 (19:00–21:30). The experiments began in August 2011 and ended in September 2011. The background noise level was 37–39 decibels.

#### Experiment and datasets

**Figure 2:**
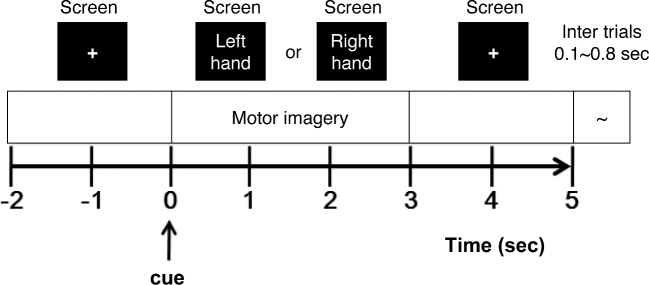
Experimental paradigm. One trial of the MI experiment.

For each subject, we recorded data for non-task-related and task (MI)-related states, as follows:

- Six types of non-task-related data: We recorded 6 types of noise data (eye blinking, eyeball movement up/down, eyeball movement left/right, head movement, jaw clenching, and resting state) for 52 subjects. Each type of noise was collected twice for 5 seconds, except the resting state, which was recorded for 60 seconds.
- Real hand movement: Before beginning the motor imagery experiment, we asked subjects to conduct real hand movements. Subjects sat in a chair with armrests and watched a monitor. At the beginning of each trial, the monitor showed a black screen with a fixation cross for 2 seconds; the subject was then ready to perform hand movements (once the black screen gave a ready sign to the subject). As shown in Fig. 2, one of 2 instructions (“left hand” or “right hand”) appeared randomly on the screen for 3 seconds, and subjects were asked to move the appropriate hand depending on the instruction given. After the movement, when the blank screen reappeared, the subject was given a break for a random 4.1 to 4.8 seconds. These processes were repeated 20 times for one class (one run), and one run was performed.
- MI experiment: The MI experiment was conducted with the same paradigm as the real hand movement experiment. Subjects were asked to imagine the hand movement depending on the instruction given. Five or six runs were performed during the MI experiment. After each run, we calculated the classification accuracy over one run and gave the subject feedback to increase motivation. Between each run, a maximum 4-minute break was given depending on the subject's demands.

The entire procedure of the experiment is presented in Table 1.

**Table 1:** Experimental procedure.

		Duration
Number	Task	(min)
1	Filling in a consent form and questionnaire	10
2	EEG electrode placement	20
3	Acquisition of the 6 types of non-task-related data	2
4	Practicing actual finger movements	3
5	RUN 1	6
6	Filling out questionnaire	4
7	RUN 2	6
8	Filling out questionnaire	4
9	RUN 3	6
10	Filling out questionnaire	4
11	RUN 4	6
12	Filling out questionnaire	4
13	RUN 5	6
14	Filling out questionnaire	4
15	Online experiment	6
16	Digitizing 3D coordinates of EEG electrodes	15
17	Removing electrodes and cleaning laboratory	20
Sum		126

#### Motor imagery instructions

**Figure 3:**
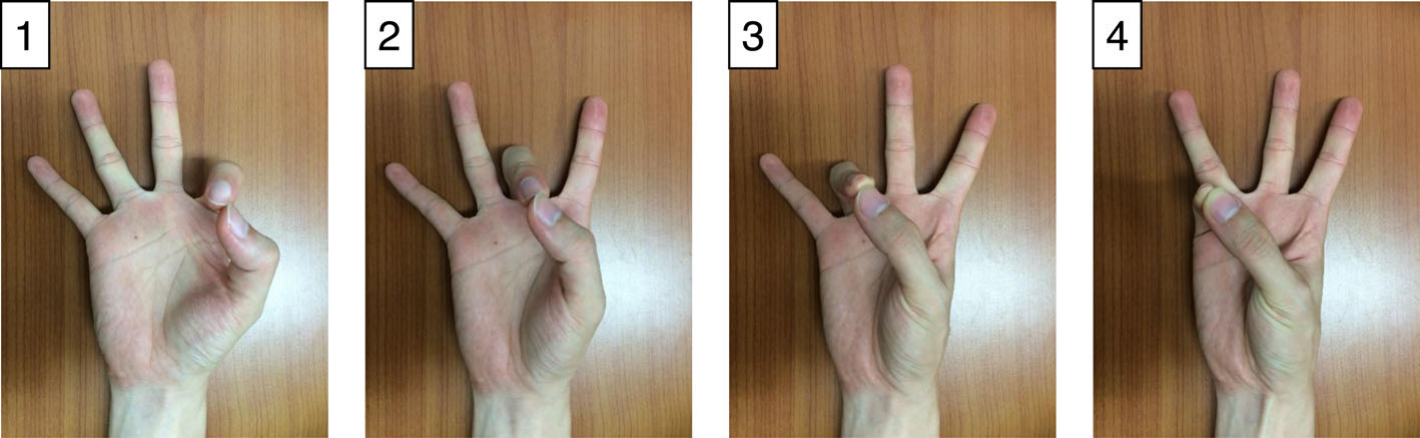
Motor imagery instruction. We asked subjects to imagine four actual finger movements: touching each index, middle, ring, and little finger to the thumb within 3 seconds. Before the MI experiment began, subjects practiced executing the four movements within 3 seconds.

Before the MI experiment began, we asked each subject to move his/her fingers, starting from the index finger and proceeding to the little finger (depicted in Fig. 3) and touching each to their thumb within 3 seconds after onset. Each subject practiced these actual finger movements, and then performed the MI experiment. When imagining the movement, we asked subjects to imagine the kinesthetic experience [8], rather than imagining the visual experience.

#### Questionnaire

We asked subjects to fill out a questionnaire during the MI experiment, as shown in Table 2. Before beginning the MI experiment, subjects answered 15 questions (questions numbered 101 to 115). After every run, subjects answered another 10 questions (questions numbered 210 to 219). After the MI experiment, we asked the subjects to answer a final set of questions (questions numbered 301 to 304). All numerical values of the questions were stored as a Microsoft Excel file (*.xlsx).

**Table 2:** Questionnaire for motor imagery experiment.

	Questionnaire								
Number	Individual information			Subject ID					
101	Time slot (1 = 9:30/2 = 12:30/3 = 15:30/4 = 19:00)								
102	Handedness (0 = left/1 = right/2 = both)								
103	Age (number)								
104	Sex (female = 0/male = 1)								
105	BCI experience (0 = no/number = how many times)								
106	Biofeedback experience (0 = no/number = how many times)								
	Before motor imagery experiment								
107	3. How long did you sleep? (1 = less than 4 h/2 = 5 ∼ 6 h/3 = 6 ∼ 7 h/4 = 7 ∼ 8 h/5 = more than 8 h)								
108	4. Did you drink coffee within the past 24 hours? (0 = no, number = hours before)								
109	5. Did you drink alcohol within the past 24 hours (0 = no, number = hours before)								
110	6. Did you smoke within the past 24 hours (0 = no, number = hours before)								
111	7. How do you feel?	Relaxed	1	2	3	4	5	Anxious	
112		Excited	1	2	3	4	5	Bored	
113	Physical state	Very good	1	2	3	4	5	Very bad or tired	
114	Mental state	Very good	1	2	3	4	5	Very bad or tired	
115	8. BCI performance (accuracy) expected? (%)								
	During motor imagery experiment								
	Run 1 (after the first run)								
210	1. Can you continue to the next run? (0 = no/1 = yes)								
211	2. How do you feel?	Relaxed	1	2	3	4	5	Anxious	
212		Excited	1	2	3	4	5	Bored	
213	Attention level	High	1	2	3	4	5	Low	
214	Physical state	Very good	1	2	3	4	5	Very bad or tired	
215	Mental state	Very good	1	2	3	4	5	Very bad or tired	
216	3. Have you nodded off (slept awhile) during this run? (0 = no/number = how many times)								
217	4. Was it easy to imagine finger movements?	Easy	1	2	3	4	5	Difficult	
218	5. How many trials did you miss? (0 = none/number = how many times)								
219	6. BCI performance (accuracy) expected? (%)								
	Run 2 (after the second run)								
220 ∼ 229			**…**						
	Run 3 (after the third run)								
230 ∼ 239			**…**						
	Run 4 (after the fourth run)								
240 ∼ 249			**…**						
	Run 5 (after the fifth run)								
250 ∼ 259			**…**						
	After the motor imagery experiment								
301	1. How was this experiment?	Duration	Short	1	2	3	4	5	Long
302		Procedure	Good	1	2	3	4	5	Bad
303		Environment	Comfortable	1	2	3	4	5	Uncomfortable
304	2. BCI performance (accuracy) of whole data expected? (%)								

#### Data format and structure

The MATLAB structure of the EEG (1st to 64th channel) and EMG (65th to 68th channel) data (“*.mat”) for each subject is shown below:

- rest: resting state with eyes-open condition
- noise:
  - eye blinking, 5 seconds × 2
  - eyeball movement up/down, 5 seconds × 2
  - eyeball movement left/right, 5 seconds × 2
  - jaw clenching, 5 seconds × 2
  - head movement left/right, 5 seconds × 2
- imagery_left: 100 or 120 trials of left hand MI
- imagery_right: 100 or 120 trials of right hand MI
- n_imagery_trials: 100 or 120 trials for each MI class
- imagery_event: value “1” represents onset for each MI trial
- movement_left: 20 trials of real left hand movement
- movement_right: 20 trials of real right hand movement
- n_movement_trials: 20 trials for each real hand movement class
- movement_event: value “1” represents onset for each movement trial
- frame: temporal range of a trial in milliseconds
- srate: sampling rate
- senloc: 3D sensor locations
- psenloc: sensor location projected to unit sphere
- subject: subject's two-digit ID - “s#”
- comment: comments for the subject
- bad_trial_indices
  - bad trials determined by voltage magnitude
  - bad trials correlated with EMG activity

### Reliability

#### Methods

For preprocessing, we used Butterworth filtering with fourth order for high-pass and band-pass filtering. We validated the EEG datasets in three different ways:

- First, we checked the number of bad trials in each subject's data. If a band-passed (8–30 Hz) trial had an amplitude greater than ±100 μV [9–11] within 500–2500 msec, the trial was declared bad. The frequency band is involved in somatosensory rhythm (SMR) [1, 12, 13]. The time window was determined by an algorithm for selection of a discriminative time interval (see the Appendix in [13]). The percentage of bad trials was estimated for each subject. The bad trials were not considered in the following analysis. The bad trial indexes were added for each subject dataset, as shown in the section titled “Data format and structure.”
- Second, we investigated whether each trial is correlated with EMG (e.g., real hand movement) adopting Vaughan and colleagues’ [14] 1998 idea, which was using correlation between class labels and EMG activity. In the prescreening of EMG in the real hand movement experiment, we observed high-frequency activity (50–250 Hz) during real hand movement. We calculated Pearson correlation between the ranked EMG power of high-frequency activity and the label of time points, as follows:
  - High-pass filtering of all EMG trials above 0.5 Hz to remove drifts;
  - Common average reference;
  - Band-pass filtering of all trials with 50–250 Hz;
  - Hilbert transform;
  - Take absolute and squared magnitudes for each complex value of all trials;
  - Extract data in resting window (−1000–0 msec) and task-related window (0–3000 msec) for each trial;
  - Prepare labels for each time point within a trial:
    * Tag “−1” value for time points in resting window;
    * Tag “+1” value for time points in task-related window;
  - Both squared EMG magnitudes and label of time points are decimated (averaged) by a factor of 8. Then calculate Pearson correlation between ranked squared EMG magnitudes and label of time points;
  - Execute permutation test over time points within a trial:
  - Calculate Pearson correlation between ranked permuted features and labels;
  - Repeat 100 times;
  - Make probability density function (PDF) of the values of Pearson correlation;
  - Calculate *P-*values (one right-tailed test) over all trials and four EMG channels;
  - If Bonferroni-corrected *P-*value is smaller than 0.01 and the correlation value is greater than 0.8 (empirically determined from real hand movement EMG data), then it is declared a bad trial correlated with EMG.
  Finally, the EMG-correlated EEG trial indices were added for each subject dataset, as shown in the “Data format and structure” section.
- Third, we checked event-related desynchronization/synchronization (ERD/ERS) of SMR for each subject [1]. To calculate ERD/ERS for each channel, we followed the same procedure as that in [1], as follows:
  - High-pass filtering of all EEG trials above 0.5 Hz to remove drifts;
  - Laplacian filtering;
  - Band-pass filtering of all trials with 8–14 Hz;
  - Hilbert transform of all trials;
  - Absolute magnitude taken for each complex value of all trials;
  - Magnitude of Hilbert-transformed samples averaged across all trials;
  - Baseline correction for each trial to obtain a percentage value for ERD/ERS per the formula ${\rm{ERD\% \ }} = \frac{{{\rm{A}} - {\rm{R}}}}{{\rm{R}}}{\rm{\ }} \times {\rm{\ }}100$, where A is each time sample and R is the mean value of the baseline period (−500 to 0 msec).
- Last, we validated the discriminability of the left versus right hand MI EEG data as classification accuracy. All trials for each subject were pre-processed by high-pass filtering and common average reference, and then filtered both spectrally (8–30 Hz) and temporally (0.5–2.5 seconds after stimulus onset). For the feature extraction algorithm, we used 2 spatial filters of the common spatial pattern (CSP) for each class [12, 13]. For classification, we used Fisher's linear discriminant analysis (FLDA). We performed cross-validation in the following way. For each class, we divided all trials of MI data into 10 subsets each. Seven subsets were chosen randomly and used to train CSP and FLDA, and the remaining 3 subsets were used to test them. This procedure was repeated 120 times by choosing 3 among the 10 subsets randomly. Finally, 120 classification accuracies were estimated and averaged.

#### Results

**Figure 4:**
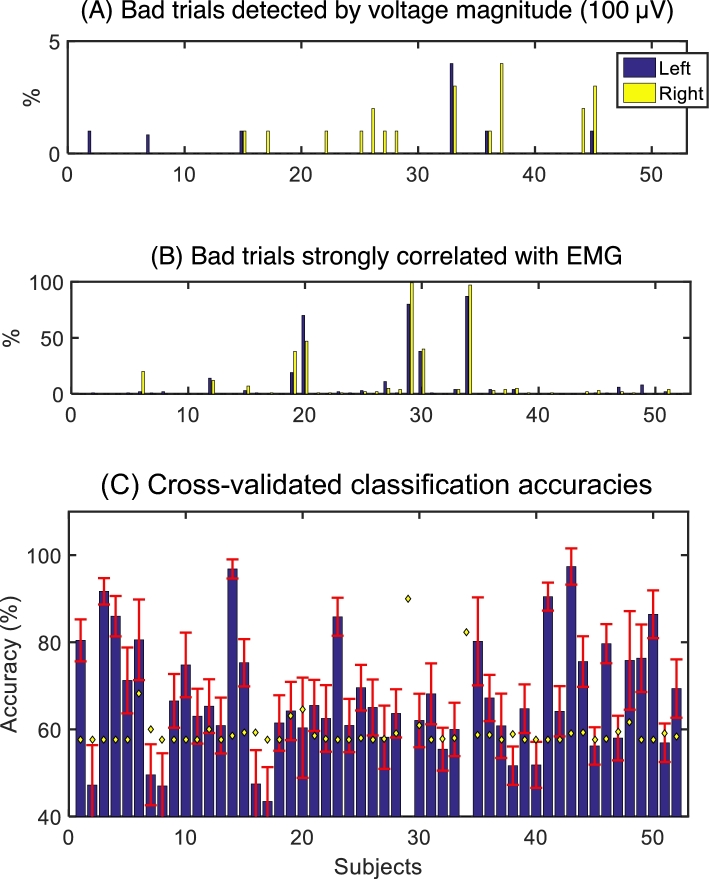
Estimated percentage of bad trials and classification accuracies for all subjects. **(A)** Each class contained 100 or 120 trials. If any amplitude in 8–30 Hz band-passed trial was greater than 100 μV within 500–2500 msec, then the trial was classified as a bad trial. **(B)** If a power level of 50–25 0Hz in EMG had correlations with the labels of resting and task-related time points within a motor imagery trial and the correlations were greater than 0.8 (correlation threshold estimated from real hand movement EMG data), then it was declared a bad trial correlated with EMG. **(C)** Cross-validated classification accuracies were estimated by using Common Spatial Pattern (CSP) and Fisher's linear discriminant analysis (FLDA). Yellow diamonds indicate the random chance levels depending on the number of total trials (excluding bad trials) for each subject.

Percentage of bad trials. We calculated the percentage of bad trials for each subject, as shown in Fig. 4A. The percentages of bad trials within the spectral and temporal discriminative ranges were below 5% for most subjects. Furthermore, we calculated the percentage of EMG trials correlated with labels of time points for each MI trial, as shown in Fig. 4B. Two subjects (s29 and s34) showed that more than 90% of their trials were correlated with EMG; most of the trials demonstrated a greater power of high-frequency EMG (50–250 Hz) in the task-related period after onset than the resting period before onset. Thus, these two subjects were declared bad subjects and were discarded in the further analysis. Rest of subjects has at least 10 trials per class. The literature [9] showed that the upper confidence limits of chance with α = 5% were 70% (classification accuracy) in a 2-class problem, with 10 trials for each class. If a subject had higher accuracy than the random chance level depending on the number of trials [9], we classified the subject into the discriminative group. On the other hand, we applied the same method to real hand movement trials to test our method. We observed that most trials (more than 85%) were correlated to the power of high-frequency EMG (50–250 Hz) and the correlation values were higher than 0.8. Here, although we set the *P-*value threshold at 0.05, a few trials were not correlated with the labels of resting or task-related states. Thus, our threshold of *P-*values was set at 0.01. Furthermore, according to the observed correlation distributions of real hand movement data, we set 0.8 as a correlation threshold. Finally, if the correlation value is greater than the 0.8 threshold and the *P-*value is smaller than 0.01 in MI datasets, the trial was classified as a bad trial correlated with real hand movement.

Most existing studies detected EMG activity through manual monitoring. They recorded EMG and EEG simultaneously and monitored EMG burst during the experiment. On the other hand, in the published literature [15], the resting state of EMG was recorded and the significant threshold from the resting EMG was defined. Furthermore, in other work in the literature [14], correlation values between target position (cursor movement control application in BCI) and EMG activity were calculated, and they were compared with the correlation values of EMG class labels and EEG class labels. Also, according to the literature [15], t-values between the EMGs of the operant hand and the non-operant hand were calculated. We believed that the correlation between EMG activity and time point labels within a trial could provide the solution for detecting which EEG trial was correlated to EMG. We attempted the voltage thresholding method, but there were trials correlated with EMG, and even EMG activity was smaller thanthe threshold. We also tried to compare the voltage distribution between the resting state and task-related EMG, but there were trials correlated to EMG although the distribution of EMG of a trial is similar to that of the resting state. Finally, 38 subjects had higher classification accuracy than their own random chance (yellow diamond marker), with a confidence level of α = 5%, as shown in Fig. 4C.

**Figure 5:**
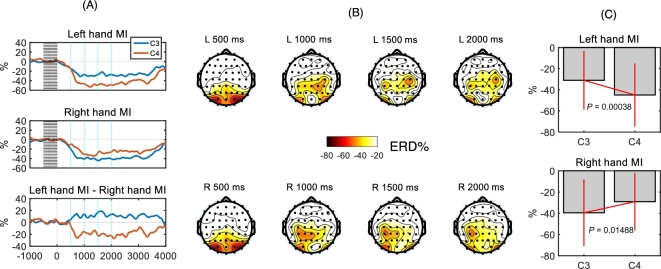
Event-related desynchronization of somatosensory rhythm (8–14 Hz) from discriminative subjects (38 subjects). **(A)** The first and second rows show ERD of the C3 and C4 channels in left and right hand motor imagery, respectively. The last row shows the difference in ERDs between left and right motor imagery. The gray shaded region is the baseline period. Cyan-colored vertical lines represent time points such as 500, 1000, 1500, and 2000 msec. **(B)** Topographies of ERDs at the cyan-colored time points in (A). Initials “L” and “R” indicate left and right motor imagery movements, respectively. **(C)** Comparison of ERD at C3 and C4 channels within 500–2500 msec. *P-*values were estimated by paired t-test.

**Figure 6:**
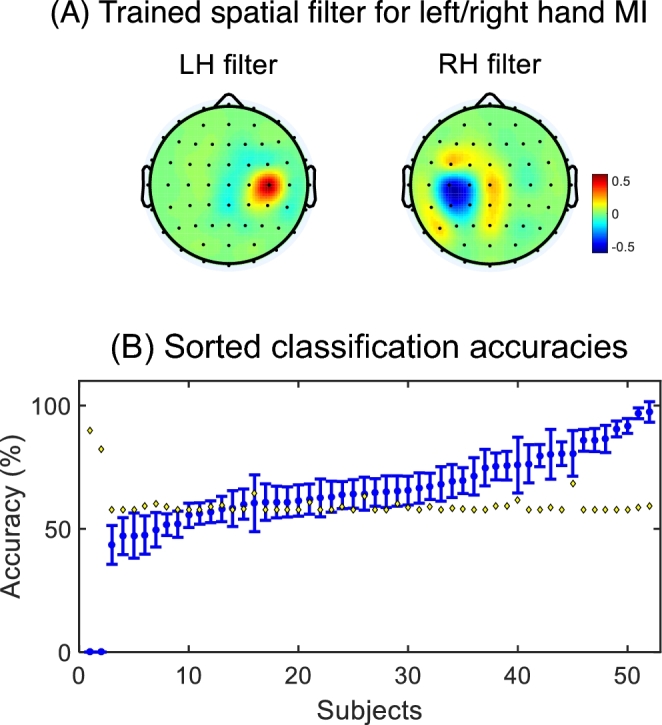
Trained spatial filters for left and right hand motor imagery data and sorted cross-validated classification results. **(A)** To demonstrate the discriminative feature of our dataset, CSP filters were trained by averaged covariance matrix of 38 subjects who had high BCI performance (>random chance). **(B)** Sorted accuracies are depicted in increasing order. Fourteen subjects showed low BCI performance (<random chance marked with yellow diamond). Because of sorting, the number on the x-axis does not correspond to subject numbers “s01” to “s52.”

ERD/ERS. The ERD/ERS results of mu rhythm (8–14 Hz) are depicted in Fig. 5. Figure 5A shows the grand averaged ERD/ERS (%) ofthe C3 and C4 channel over the 36 subjects who had discriminative information (as shown in Fig. 4C). The powers of mu rhythm in the C3 and C4 channel decreased in both left and right hand MI. The contralateral channel showed bigger desynchronization in the corresponding class. The last row in Fig. 5A shows the difference over time of ERD/ERS in the C3 and C4 channels. The C4 channel showed a bigger difference than the C3 channel. Fig. 5B shows the topographies at specific time points, for instance, 500, 1000, 1500, and 2000 msec. Those time points are marked in Fig. 5A as a cyan-colored vertical line. At 500 msec, the occipital areas were activated (“alpha inhibition” [1, 16]). The occipital alpha ERD was continued to 2000 msec. We also tested whether each trial contains occipital alpha ERD or not by using permutation test, just like EMG trial detection. We found that most trials have the occipital alpha ERD for all subjects. Common spatial pattern (CSP) filters (representing the filtering weight for each channel) trained by the 38 subjects were estimated, as shown in Fig. 6A; prominent CSP weights existed around SMC only, but not in occipital. It means that the occipital alpha ERD may not affect the class-labeled data. It is expected that the activation of the occipital area may be related to processing of visual stimulus. However, the occipital alpha activity was not influenced on the quality of our left/right motor imagery data. At 1000 msec, contralateral channels showed bigger ERD than ipsilateral channels. For left hand MI, the right central and parietal areas showed bigger ERD than the left hemisphere. In Fig. 5C, bar graphs of left hand MI show that the contralateral ERD (C4 channel) is stronger than the ipsilateral ERD (C3 channel).

Classification. The mean accuracy of all BCI performances (Fig. 4C) over the 50 subjects, excluding bad subjects, was 67.46% (±13.17%) in our datasets. In BCI2000 MI datasets [17–19], the average accuracy was 60.42% (±11.68%) over 109 subjects using CSP and FLDA [20]. In our datasets, 14 subjects (26.92% of 52 subjects) showed low BCI performance (below random chance, which is the upper confidence limit of chance with α = 5%), as shown in Fig. 6B. This is greater than a report on 99 subjects [21] showing that 6.7% of the subjects had accuracies lower than 60% (here, the average accuracy over the 99 subjects was not reported). Comparing with the datasets of [19], our datasets have more trials, even though bad trials were rejected and excluded from the results. The EEG Motor Movement/Imagery Dataset [19] has MI data of 109 subjects, but the number of total trials for each subject is about 20 trials, which has a random chance level of 65% (α = 5%).

## Abbreviations

BCI: brain–computer interface; CSP: common spatial pattern; EEG: electroencephalography; EMG: electromyography; ERD/ERS: event-related desynchronization/synchronization; FLDA: Fisher's linear discriminant analysis; MI: motor imagery; SMR: somatosensory rhythm.

## Acknowledgments

This work was supported by GIST Research Institute (GRI) grant funded by the GIST in 2017, and Institute for Information & Communication Technology Promotion (IITP) grant funded by the Korea government (No. 2017-0-00451).

## Availability of supporting data

The data supporting this paper, including EEG datasets and questionnaire results, are available in the *GigaScience* database, GigaDB [6].

## Competing interests

The authors declare that they have no competing interests.

## Supplementary Material

GIGA-D-16-00104_Original_Submission.pdf

GIGA-D-16-00104_Revision_1.pdf

GIGA-D-16-00104_Revision_2.pdf

GIGA-D-16-00104_Revision_3.pdf

Response_to_reveiwer_comments_Original_Submission.pdf

Response_to_reviewer_comments_revision_1.pdf

Response_to_reviewer_comments_revision_2.pdf

Reviewer_1_Report_(Original_Submission).pdf

Reviewer_1_Report_(revision_1).pdf

Reviewer_2_Report_(Original_Submission).pdf

## References

[1] Pfurtscheller G, Lopes da Silva FH Event-related EEG/MEG synchronization and desynchronization: basic principles. Clin Neurophysiol 1999;110:1842–57. 10576479 10.1016/s1388-2457(99)00141-8

[2] Grosse-Wentrup M, Schölkopf B A review of performance variations in SMR-based brain-computer interfaces (BCIs). In: Guger C, Allison BZ, Edlinger G, eds. Brain–Computer Interface Research. New York: Springer; 2013:39–51. http://link.springer.com/chapter/10.1007/978-3-642-36083-1_5 (21 December 2016, date last accessed).

[3] Blankertz B, Sannelli C, Halder S Neurophysiological predictor of SMR-based BCI performance. Neuroimage 2010;51:1303–9. 20303409 10.1016/j.neuroimage.2010.03.022

[4] Ahn M, Cho H, Ahn S High theta and low alpha powers may be indicative of BCI-Illiteracy in motor imagery. PLoS One 2013;8:e80886. 24278339 10.1371/journal.pone.0080886 PMC3838377

[5] Cho H, Ahn M, Kim K Increasing session-to-session transfer in a brain–computer interface with on-site background noise acquisition. J Neural Eng 2015;12:66009. 10.1088/1741-2560/12/6/066009 26447843

[6] Cho H, Ahn M, Ahn S Supporting data for “EEG datasets for motor imagery brain computer interface.” GigaScience Database 2017; http://dx.doi.org/10.5524/100295. 10.1093/gigascience/gix034 PMC5493744 28472337

[7] Schalk G, McFarland DJ, Hinterberger T BCI2000: a general-purpose brain-computer interface (BCI) system. IEEE Trans Biomed Eng 2004;51:1034–43. 15188875 10.1109/TBME.2004.827072

[8] Neuper C, Scherer R, Reiner M Imagery of motor actions: Differential effects of kinesthetic and visual–motor mode of imagery in single-trial EEG. Cogn Brain Res 2005;25:668–77. 10.1016/j.cogbrainres.2005.08.014 16236487

[9] Müller-Putz G, Scherer R, Brunner C Better than random: a closer look on BCI results. Int J Bioelectromagn 2008;10:52–5.

[10] Muthukumaraswamy S High-frequency brain activity and muscle artifacts in MEG/EEG: a review and recommendations. Front Hum Neurosci 2013;7:138. 23596409 10.3389/fnhum.2013.00138 PMC3625857

[11] van Dinteren R, Arns M, Jongsma ML P300 development across the lifespan: a systematic review and meta-analysis. PLoS One 2014;9:e87347. 24551055 10.1371/journal.pone.0087347 PMC3923761

[12] Ramoser H, Muller-Gerking J, Pfurtscheller G Optimal spatial filtering of single trial EEG during imagined hand movement. IEEE Trans Rehabil Eng 2000;8:441–6. 11204034 10.1109/86.895946

[13] Blankertz B, Tomioka R, Lemm S Optimizing spatial filters for robust EEG single-trial analysis. IEEE Signal Process Mag 2008;25:41–56.

[14] Vaughan TM, Miner LA, McFarland DJ EEG-based communication: analysis of concurrent EMG activity. Electroencephalogr Clin Neurophysiol 1998;107:428–33. 9922089 10.1016/s0013-4694(98)00107-2

[15] Matsumoto J, Fujiwara T, Takahashi O Modulation of mu rhythm desynchronization during motor imagery by transcranial direct current stimulation. J Neuroengineering Rehabil 2010;7:1. 10.1186/1743-0003-7-27 PMC2898754 20540721

[16] Jensen O, Mazaheri A Shaping functional architecture by oscillatory alpha activity: gating by inhibition. Front Hum Neurosci 2010;4:186. 21119777 10.3389/fnhum.2010.00186 PMC2990626

[17] Goldberger AL, Amaral LA, Glass L Physiobank, physiotoolkit, and physionet components of a new research resource for complex physiologic signals. Circulation 2000;101:e215–20. 10851218 10.1161/01.cir.101.23.e215

[18] BCI2000 wiki. www.bci2000.org (15 April 2017, date last accessed).

[19] EEG Motor Movement/Imagery Dataset. https://physionet.org/pn4/eegmmidb/ (15 April 2017, date last accessed).

[20] Cho H, Ahn S, Jun SC How is subject-to-subject transfer probable in motor imagery BCI? In: Proceeding of the 6th International Brain-Computer Interface Meeting, 2016. Article ID: 167. Graz: Verlag der TU Graz, Graz University of Technology, 2016.

[21] Guger C, Edlinger G, Harkam W How many people are able to operate an EEG-based brain-computer interface (BCI)? IEEE Trans Neural Syst Rehabil Eng 2003;11(2):145–7. 12899258 10.1109/TNSRE.2003.814481

