# Supplementary material for: EEG datasets for motor imagery brain–computer interface
Source: Gigascience. 2017 May 4;6(7):1–8. doi: 10.1093/gigascience/gix034 (PMC5493744; doi:10.1093/gigascience/gix034)
Supplement: GIGA-D-16-00104_Original_Submission.pdf [file gix034_GIGA-D-16-00104_Original_Submission.pdf]

Manuscript
Click here to view linked References
Click here to download Manuscript GigaScience_hcho_ms_final_v1.docx

Full title:

EEG datasets for motor imagery brain computer interface

Hohyun **Cho**[1], Minkyu **Ahn**[2], Sangtae **Ahn**[1], Moonyoung **Kwon**[1], and Sung Chan **Jun**[1*]

[1] School of Electrical Engineering and Computer Science, Gwangju Institute of Science and Technology, Gwangju, Korea

[2] Department of Neuroscience, Brown University, Providence, RI, U.S.A. 02912

***** Corresponding author email: scjun@gist.ac.kr (SCJ)

Full address:

Dasan 507, Gwangju Institute of Science and Technology, 123 Cheomdangwagi-ro, Buk-gu, Gwangju 61005, South Korea
TEL: +82-062-715-2216
FAX: +82-062-715-2204

Abstract

Background: Most investigators of brain computer interface (BCI) believe that ideal BCI can be achieved by induced neuronal activity from the cortex, but not evoked neuronal activity. Motor imagery (MI) based BCI is one of the standard concepts of ideal BCI, in that the user can generate induced activity by imagining motor movements. However, variations in performance over sessions and subjects are too severe to overcome easily, and thus, a basic understanding and investigation of BCI performance variation is necessary to find critical evidence of performance variation.

Here we present not only EEG datasets for MI BCI from 52 subjects, but also the results of a psychological and physiological questionnaire, the locations of 3D EEG electrodes, and EEGs for non-task related states.

Findings: We validated our EEG datasets by using the percentage of bad trials, event-related desynchronization/synchronization (ERD/ERS) analysis, and classification analysis. After conventional rejection of bad trials, we showed contralateral ERD and ipsilateral ERS in the somatosensory area, which are well-known patterns of MI. Finally, we showed that 71.15% of datasets (37 subjects) included reasonably discriminative information, resulting in a classification accuracy of over 60% with a confidence level of α=1%.

Conclusions: Our EEG datasets included good information necessary to determine statistical significance; they consisted of well-discriminated datasets (37 subjects) and less-discriminative datasets (15 subjects). These may provide researchers with opportunities to investigate human factors related to MI BCI performance variation, and achieve subject-to-subject transfer by using metadata, including a questionnaire, EEG coordinates, and EEGs for non-task related states.

Key words

Motor imagery, EEG, brain computer interface, performance variation, subject-to-subject transfer

Data types

EEG datasets

Data size

Data size of a subject: 152 – 192 MB

Data size of 52 subjects: 8.25 GB

Readme file

Attached as following file name: “README_v1.txt”

Data description

Background and Purpose

Motor imagery (MI) based brain computer interface (BCI) is a topic that has attracted great interest. Compared with other BCI paradigms, MI BCI can provide users with direct communication without any limb movement or external stimulus (for example, P300 based BCI). Thus, MI BCI is an almost ideal form of BCI. Ideal BCI prefers “induced” brain activity [1] from the cortex, rather than “evoked” brain activity. Although the MI BCI is fascinating, it has many obstacles. Among these are the facts that BCI researchers have tended to focus on subject-to-subject transfer (training subject-independent algorithm). To achieve effect subject-to-subject transfer, it is important to understand the variation in performance over subjects. Predicting subject’s performance by using the resting state, or background noise from EEG, are some examples of this [2–4].

In this paper, we recorded MI BCI EEG datasets with 2 classes (100 trials for each class) from 52 healthy subjects. To study various forms of evidence of performance variation and subject-to-subject transfer, we collected subjective answers to a psychological and physiological questionnaire, as well as EEG. In addition, we recorded the locations of 3D EEG electrodes, and non-task related EEG (resting state, eyeball and head movements, and jaw clenching). Here, we validated our datasets using the percentage of bad trials, spectral analysis, and classification analysis. These datasets were stored in the GigaScience database, GigaDB.

Experimental design

Subjects

We conducted a BCI experiment for motor imagery movement (MI movement) of the left and right hands with 52 subjects (26 females, mean age±SD age = 24.8±3.86); the experiment was approved by the Institutional Review Board of Gwangju Institute of Science and Technology. Each subject took part in the same experiment and subject ID was denoted and indexed as s1, s2, …, s52. All subjects gave informed consent to collect information on brain signals and were paid for their participation. The data collected were used only for research purposes.

Figure 1. EEG channel configuration–numbering (left) and corresponding labeling (right).

Recording software and device

EEG data were collected from 64 Ag/AgCl active electrodes. As shown in Figure 1, a 64-channel montage based on the international 10-10 system was used to record the EEG signals with 512Hz sampling rates. The EEG device used in this experiment was the Biosemi ActiveTwo system. The BCI2000 system 3.0.2 [5] was used to collect EEG data and present instructions (left hand or right hand MI). Furthermore, we recorded EMG as well as EEG simultaneously to check actual hand movements. Two EMG electrodes were attached to the flexor digitorum profundus and extensor digitorum on each arm.

For each subject, EEG channel locations (3D coordinates) were collected with a 3D coordinate digitizer (Polhemus Fastrak). Electrode location was measured as the average of three measurements of the digitizer to obtain a stabilized position to prevent hand shaking.

Figure 2. Experimental paradigm. One trial of the MI experiment.

Environment

All experiments were conducted at our laboratory during one of four time slots: T1 (9:30-12:00), T2 (12:30-15:00), T3 (15:30-18:00), and T4 (19:00-21:30). The experiments began on August, 2011 and ended on September, 2011. The background noise level was 37-39 decibels.

Experiment and datasets

For each subject, we recorded data for non-task related and task (MI) related states, as follows:

- **Six types of non-task related data.** We recorded 6 types of noise data (eye blinking, eyeball movement up/down, eyeball movement left/right, head movement, jaw clenching, and resting state) for 52 subjects. Each type of noise was collected twice for 5 seconds, except the resting state, which was recorded for 60 seconds.

- **MI experiment.** Subjects sat in a chair with armrests and watched a monitor. At the beginning of a trial, the monitor showed a black screen with a fixation cross for 2 seconds; the subject was then ready to imagine hand movements (the black screen gives a ready sign to the subject). As shown in Figure 2, one of two instructions ("left hand" or "right hand") appeared randomly on the screen for 3 seconds, and subjects were asked to imagine the hand movement depending on the instruction given. After imagination, when the blank screen appeared, the subject was given a break for a random 4.1 to 4.8 seconds. These processes were repeated 20 times for one class (one run), and 5 runs were performed. After each run, we calculated the classification accuracy over one run and gave the subject feedback to increase motivation. Between each run, a maximum 4-minute break was given depending on the subject's demands.

The entire procedure of the experiment is presented in Table 2.

Figure 3. Motor imagery instruction. We asked subjects to imagine four actual finger

movements: touching each index, middle, ring, and little finger with the thumb within 3 seconds. Before the MI experiment began, subjects practiced executing the four movements within 3 seconds.

Motor imagery instructions

Before the MI experiment began, we asked each subject to move his/her fingers, starting from the index finger and proceeding to the little finger (depicted in Figure 3) within 3 seconds after onset. Each subject practiced these actual finger movements, and then performed the MI experiment. When imagining the movement, we asked subjects to imagine the kinesthetic experience, rather than imagining the visual experience.

Table 1. Experimental procedure.

Number	Task	Duration (min)
1	Filling in a consent form and questionnaire	10
2	EEG electrode placement	20
3	Acquisition of the six types of non-task related data	2
4	Practicing actual finger movements	3
5	RUN 1	6
6	Filling out questionnaire	4
7	RUN 2	6
8	Filling out questionnaire	4
9	RUN 3	6
10	Filling out questionnaire	4
11	RUN 4	6
12	Filling out questionnaire	4
13	RUN 5	6
14	Filling out questionnaire	4
15	Online experiment	6
16	Digitizing 3D coordinates of EEG electrodes	15
17	Removing electrodes and cleaning laboratory	20
	Sum	126

Table 2. Questionnaire for motor imagery experiment.

Questionnaire									
Number	**Individual Information**		Subject ID:						
101	Time slot (1=9:30/2=12:30/3=15:30/4=19:00)								
102	Handedness (0=left/1=right/2=both)								
103	Age (number)								
104	Sex (female=0/male=1)								
105	BCI experience (0=no/number=how many times)								
106	Biofeedback experience (0=no/number=how many times)								
	**Before motor imagery experiment**								
107	3. How long did you sleep ? (1=less than 4h/2=5~6h/3=6~7h/4 = 7~8h/5= more than 8h)								
108	4. Did you drink coffee within the past 24 hours? (0=no, number=hours before)								
109	5. Did you drink alcohol within the past 24 hours (0=no, number=hours before)								
110	6. Did you smoke within the past 24 hours (0=no, number=hours before)								
111	7. How do you feel?		Relaxed	1	2	3	4	5	Anxious
112			Excited	1	2	3	4	5	Bored
113		Physical state	Very good	1	2	3	4	5	Very bad or tired
114		Mental state	Very good	1	2	3	4	5	Very bad or tired
115	8. BCI performance (accuracy) expected? (%)								
	**During motor imagery experiment**								
	Run 1(after the first Run)								
210	1. Can you continue to the next run? (0=no/1=yes)								
211	2. How do you feel?		Relaxed	1	2	3	4	5	Anxious
212			Excited	1	2	3	4	5	Bored
213		Attention level	High	1	2	3	4	5	Low
214		Physical state	Very good	1	2	3	4	5	Very bad or tired
215		Mental state	Very good	1	2	3	4	5	Very bad or tired
216	3. Have you nodded off (slept awhile) during this run? (0=no/number = how many times)								
217	4. Was it easy to imagine finger movements?		Easy	1	2	3	4	5	Difficult
218	5. How many trials did you miss? (0=none/number = how many times)								
219	6. BCI performance (accuracy) expected? (%)								
	Run 2 (after the second Run)								
220~229			...						
	Run 3 (after the third Run)								
230~239			...						
	Run 4 (after the fourth Run)								
240~249			...						
	Run 5 (after the fifth Run)								
250~259			...						
	**After the motor imagery experiment**								
301	1. How was this experiment?	Duration	Short	1	2	3	4	5	Long
302		Procedure	Good	1	2	3	4	5	Bad
303		Environment	Comfortable	1	2	3	4	5	Uncomfortable
304	2. BCI performance (accuracy) of whole data expected? (%)								

Figure 4. Estimated percentage of bad trials and cross-validated classification accuracies for all subjects. (A) Each class had 100 trials. The maximum percentage of bad trials was 4% (s34). (B) Individual accuracies are depicted over 52 subjects.

Questionnaire

We asked subjects to fill out a questionnaire during the MI experiment, as shown in Table 1. Before beginning the MI experiment, subjects answered 15 questions (question numbers 101 to 115). After every run, subjects answered another 10 questions (question numbers 210 to 219). After the MI experiment, we asked the subjects to answer a final set of questions (question numbers 301 to 304). All numerical values of the questions were stored as an excel file (*.xlsx).

Data format and structure

The structure of the MATLAB EEG data ('*.mat') for each subject is shown below:

- Rest: eyes-open condition
- Noise:
  - Eyeball movement up/down 5 sec. × 2
  - Eyeball movement left/right 5 sec. × 2
  - Head movement up/down 5 sec. × 2
  - Head movement left/right 5 sec. × 2
  - Jaw clenching 5 sec. × 2
- Raw_left: 100 trials of left hand MI EEG
- Raw_right: 100 trials of right hand MI EEG
- Emg_left: EMG data during left hand MI
- Emg_right: EMG data during right hand MI
- N_trials: 100 trials for each class
- Frame: temporal range of a trial in milliseconds
- Srate: sampling rate
- Event: value "1" represents onset for each trial
- Senloc: 3D sensor locations
- Psenloc: sensor location projected to unit sphere
- Subject: subject two digit ID - 's##'

- Comment: comments

Figure 5. Event-related desynchronization (ERD) of somatosensory rhythm (8-30Hz). (A) Initials "L" and "R" indicate left and right hemispheres, respectively. Initials "F", "C", "P", and "O" indicate frontal, central, parietal, and occipital lobes, respectively. Blue dotted line is 500 and 700 msec. (B) Topographies of ERD within 500-700 msec. (C) Comparison of ERD at C3 and C4 channels within 500-2500 msec.

Figure 6. Sorted cross-validated classification results. Sorted accuracies are depicted in increasing order. Fifteen subjects showed low BCI performance (<60%). Red dotted lines indicate 60%. Because of sorting, the number on the X-axis does not correspond to subject numbers "s1" to "s50."

Reliability

Methods

We validated the EEG datasets in three different ways:

- Firstly, we checked the number of bad trials in each subject's data. If a band-passed (8–30 Hz) trial had an amplitude greater than ±100 μV [6,7] within 500–2500 msec, the trial was declared bad. The frequency band is involved in somatosensory rhythm (SMR) [1,8,9]. The time window was determined by an algorithm for selection of a discriminative time interval [9] (see appendix). The percentage of bad trials was estimated for each subject. The bad trials were not considered in the following analysis.
- Secondly, we checked event-related desynchronization/synchronization (ERD/ERS) of SMR for each subject [1]. To calculate ERD/ERS for each channel, we followed the same procedure as that in [1], as follows:
    1. Band-pass filtering of all trials with 8-30 Hz;
    2. Hilbert transform of all trials;
    3. Absolute magnitude taken for each complex value of all trials;
    4. Magnitude of Hilbert transformed samples averaged across all trials;
    5. Baseline correction for each trial to obtain a percentage value for ERD/ERS according to the formula $\mathrm{ERD\%} = \frac{\mathrm{A-R}}{\mathrm{R}} \times 100$, where A is each time sample and R is the mean value of the baseline period (-500 to 0 msec).
- Lastly, we validated the discriminability of the MI EEG data as classification accuracy. All trials for each subject were filtered both spectrally (8-30Hz) and temporally (0.5-2.5 seconds after stimulus onset). For the feature extraction algorithm, we used 5 spatial filters of the common spatial pattern (CSP) for each class [8,9]. For classification, we used fisher linear discriminant analysis (FLDA). We performed

cross-validation in the following way. For each class, we divided the 100 trials of MI data into 10 subsets of 10 trials each. Seven subsets were chosen randomly and used to train CSP and FLDA, and the remaining 3 subsets were used to test them. This procedure was repeated 120 times by choosing 3 among the 10 subsets randomly. Finally, 120 classification accuracies were estimated and averaged.

Results

Percentage of bad trials. We estimated the percentage of bad trials for each subject, as shown in Figure 4. The percentages of bad trials within the spectral and temporal discriminative ranges were zero for most subjects, and at most, 4% for the remaining few subjects. The literature [6] conducted a classification simulation of a 2-class problem with 80 trials for each class. The results showed that the upper confidence limits of chance with α=5% and α=1% were 57.5% and 60%, respectively. In our datasets, there were more than 80 good trials, even after we rejected bad trials. According to this reasoning, it is good to say that a subject who demonstrated more than 60% accuracy provided discriminative information for the left and right hand EEG with a confidence level of α=1% and had the potential to improve his/her classification accuracy.

ERD/ERS. The ERD/ERS results are depicted in Figure 5. Figure 5A shows the grand averaged ERD/ERS (%) over the 52 subjects. For better representation, we clustered 64 channels into 8 groups of channels: LF/RF (left/right frontal), LC/RC (left/right central), LP/RP (left/right parietal), and LO/RO (left/right occipital). Furthermore, we plotted topography and a bar graph of the intensive ERD period (500-700 msec), as shown in Figures 5B and C. Topographies show that the central, parietal, and occipital areas are involved in the MI task. Bar graphs of the left hand MI show that the contralateral ERD (C4 channel) is stronger than the ipsilateral ERD (C3 channel).

Classification. The mean accuracy of all BCI performances (Figure 4B) over the 52 subjects was 67.68% (±12.23%) in our datasets, while in BCI2000 MI datasets [5,10,11], the average accuracy was 60.42% (±11.68%) over 109 subjects using CSP and FLDA [12]. In our datasets, fifteen subjects (28.85% of 52 subjects) showed low BCI performance (below 60% accuracy, which is the upper confidence limit of chance with α=1%), as shown in Figure 6. This is greater than a report on 99 subjects [13] that showed that 6.7% of the subjects had accuracies lower than 60% (here, the average accuracy over the 99 subjects was not reported).

Availability of supporting data

Supporting data in this paper can be found in the GigaScience database, GigaDB.

Abbreviations

BCI: Brain Computer Interface; EEG: Electroencephalography; ERD/ERS: Event-related desynchronization/synchronization; SMR: Somatosensory Rhythm; MI: motor imagery;

Competing interests

The authors declare that they have no competing interests.

Authors' contributions

MA and HC designed the experiments. HC, SA, MK, and MA conducted the experiments. HC

and MA designed the data validation method and analyzed the data. HC prepared the datasets for storage. SCJ coordinated all experiments. SCJ and HC wrote the paper. All authors read and approved the final manuscript.

Acknowledgements

This work was supported by the Ministry of Culture, Sports and Tourism (MCST) and the Korea Creative Content Agency (KOCCA) in the Culture Technology (CT) Research & Development Program 2016.

References

1. Pfurtscheller G, Lopes da Silva FH. Event-related EEG/MEG synchronization and desynchronization: basic principles. Clin. Neurophysiol. 1999;110:1842–57.

2. Blankertz B, Sannelli C, Halder S, Hammer EM, Kübler A, Müller K-R, et al. Neurophysiological predictor of SMR-based BCI performance. NeuroImage. 2010;51:1303–9.

3. Ahn M, Cho H, Ahn S, Jun SC. High Theta and Low Alpha Powers May Be Indicative of BCI-Illiteracy in Motor Imagery. PLoS ONE. 2013;8:e80886.

4. Cho H, Ahn M, Kim K, Jun SC. Increasing session-to-session transfer in a brain–computer interface with on-site background noise acquisition. J. Neural Eng. 2015;12:66009.

5. Schalk G, McFarland DJ, Hinterberger T, Birbaumer N, Wolpaw JR. BCI2000: a general-purpose brain-computer interface (BCI) system. IEEE Trans. Biomed. Eng. 2004;51:1034–43.

6. Daly I, Pichiorri F, Faller J, Kaiser V, Kreilinger A, Scherer R, et al. What does clean EEG look like? 2012 Annu. Int. Conf. IEEE Eng. Med. Biol. Soc. 2012. p. 3963–6.

7. Muthukumaraswamy S. High-frequency brain activity and muscle artifacts in MEG/EEG: a review and recommendations. Front. Hum. Neurosci. 2013;7:138.

8. Ramoser H, Muller-Gerking J, Pfurtscheller G. Optimal spatial filtering of single trial EEG during imagined hand movement. IEEE Trans. Rehabil. Eng. 2000;8:441–6.

9. Blankertz B, Tomioka R, Lemm S, Kawanabe M, Muller K-R. Optimizing Spatial filters for Robust EEG Single-Trial Analysis. IEEE Signal Process. Mag. 2008;25:41–56.

10. Goldberger AL, Amaral LA, Glass L, Hausdorff JM, Ivanov PC, Mark RG, et al. Physiobank, physiotoolkit, and physionet components of a new research resource for complex physiologic signals. Circulation. 2000;101:e215–e220.

11. BCI2000 wiki [Internet]. Available from: www.bci2000.org

12. Cho H, Ahn S, Jun SC. How is subject-to-subject transfer probable in motor imager BCI? Proceeding Sixth Int. Brain-Comput. Interface Meet. 2016;Article ID: 167.

13. Guger C, Edlinger G, Harkam W, Niedermayer I, Pfurtscheller G. How many people are able to operate an EEG-based brain-computer interface (BCI)? IEEE Trans. Neural Syst. Rehabil. Eng. Publ. IEEE Eng. Med. Biol. Soc. 2003;11:145–7.

Figure

Figure 1

Click here to download Figure figures.pptx

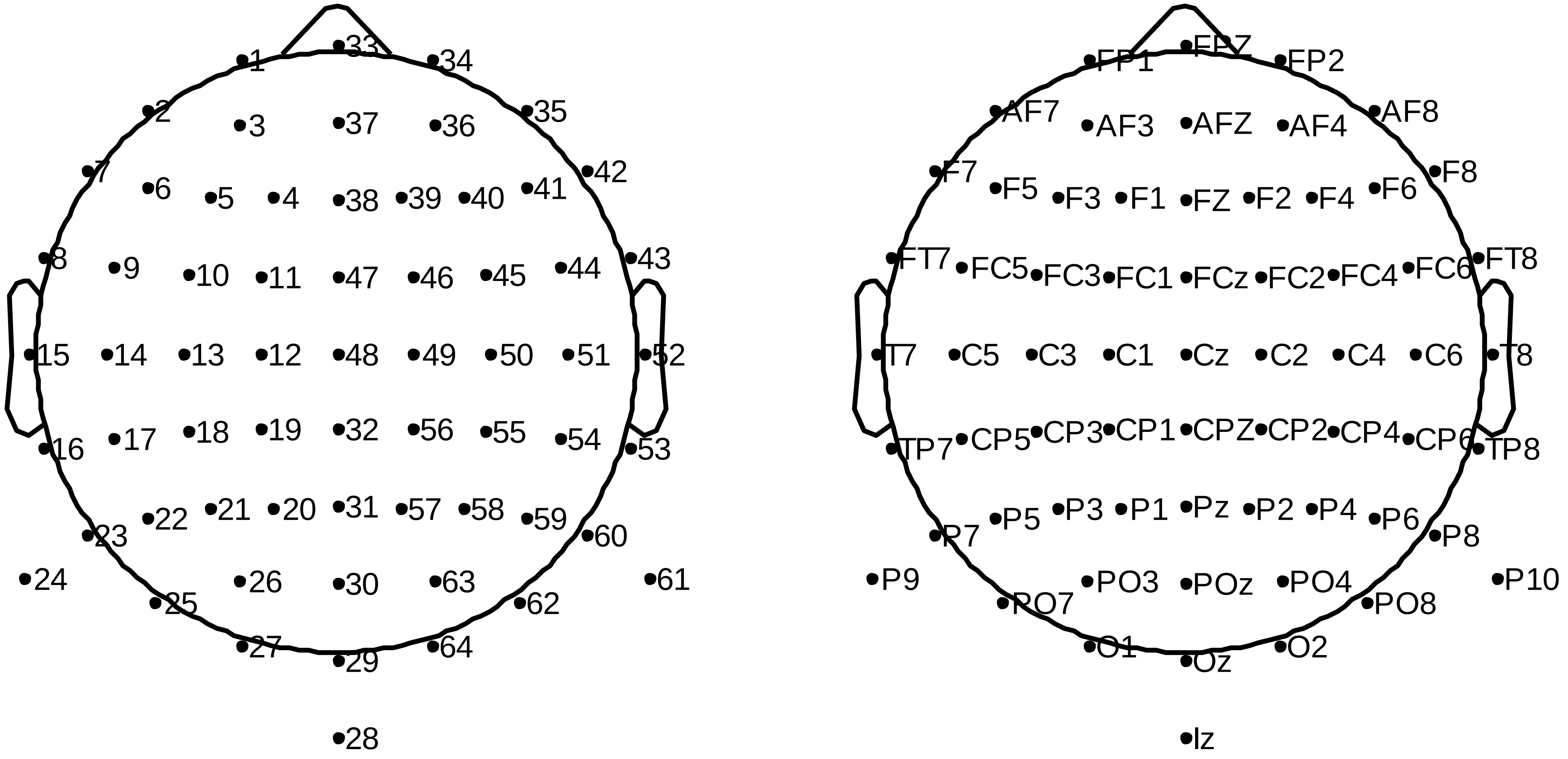

Figure 2

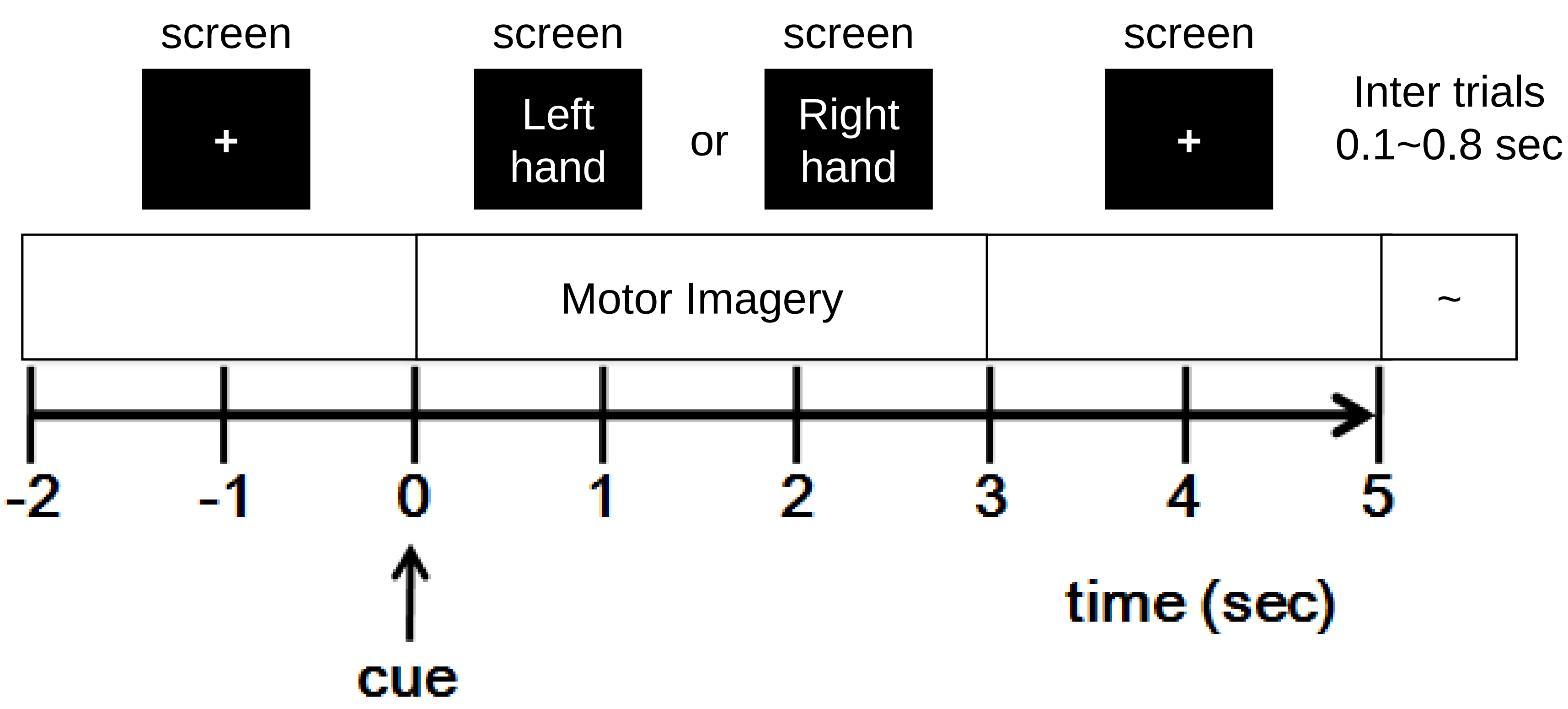
screen
screen
screen
screen
+
Left hand
or
Right hand
+
Inter trials 0.1~0.8 sec
Motor Imagery
~
-2
-1
0
1
2
3
4
5
cue
time (sec)

Figure 3

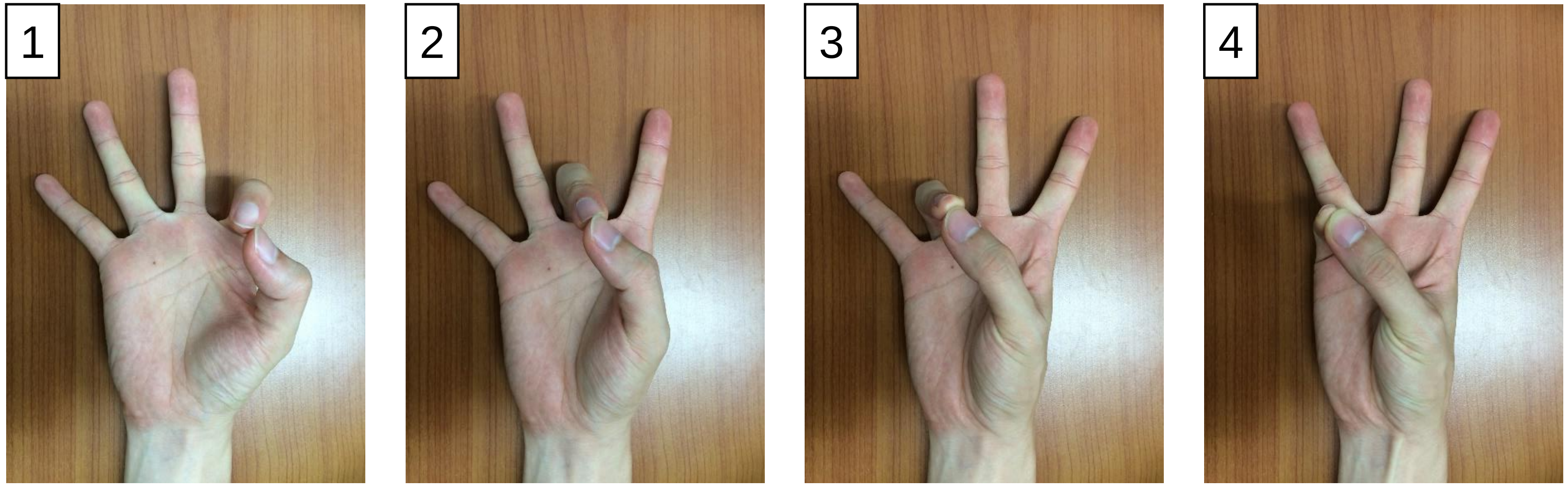

Figure 4A

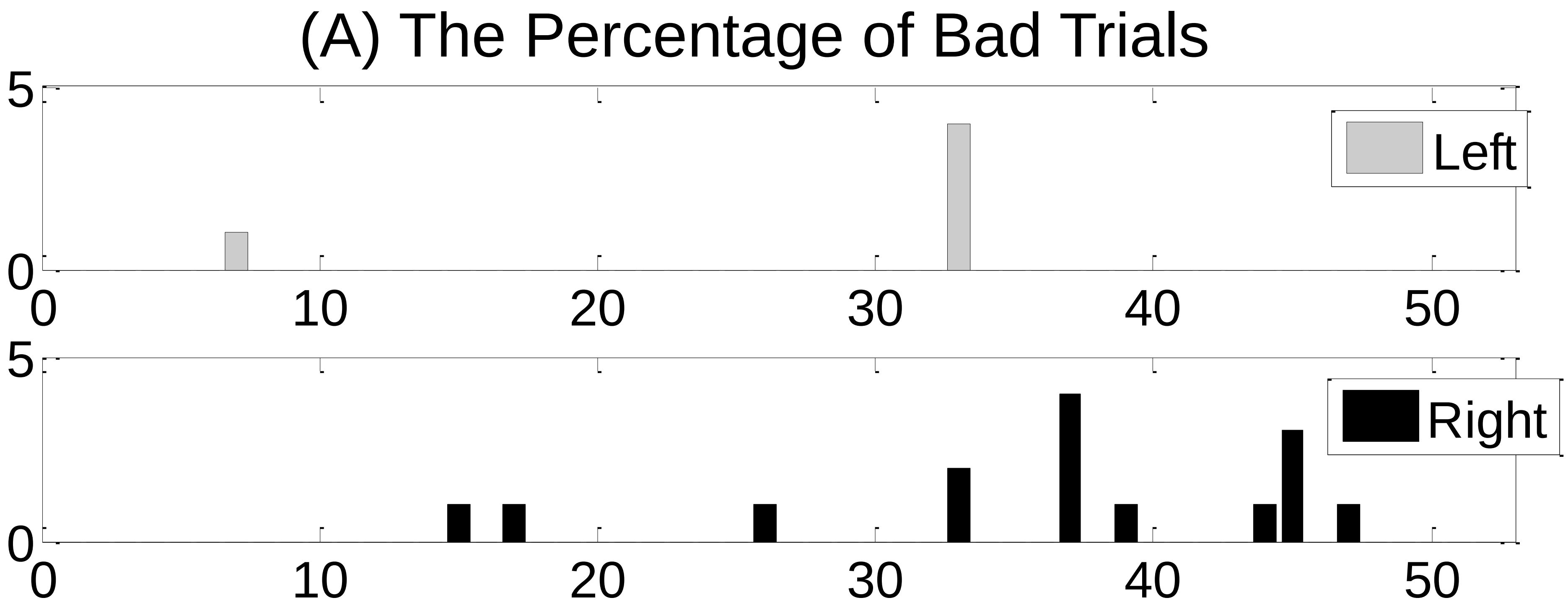
(A) The Percentage of Bad Trials
5
0
0
10
20
30
40
50
Left
5
0
0
10
20
30
40
50
Right

Figure 4B

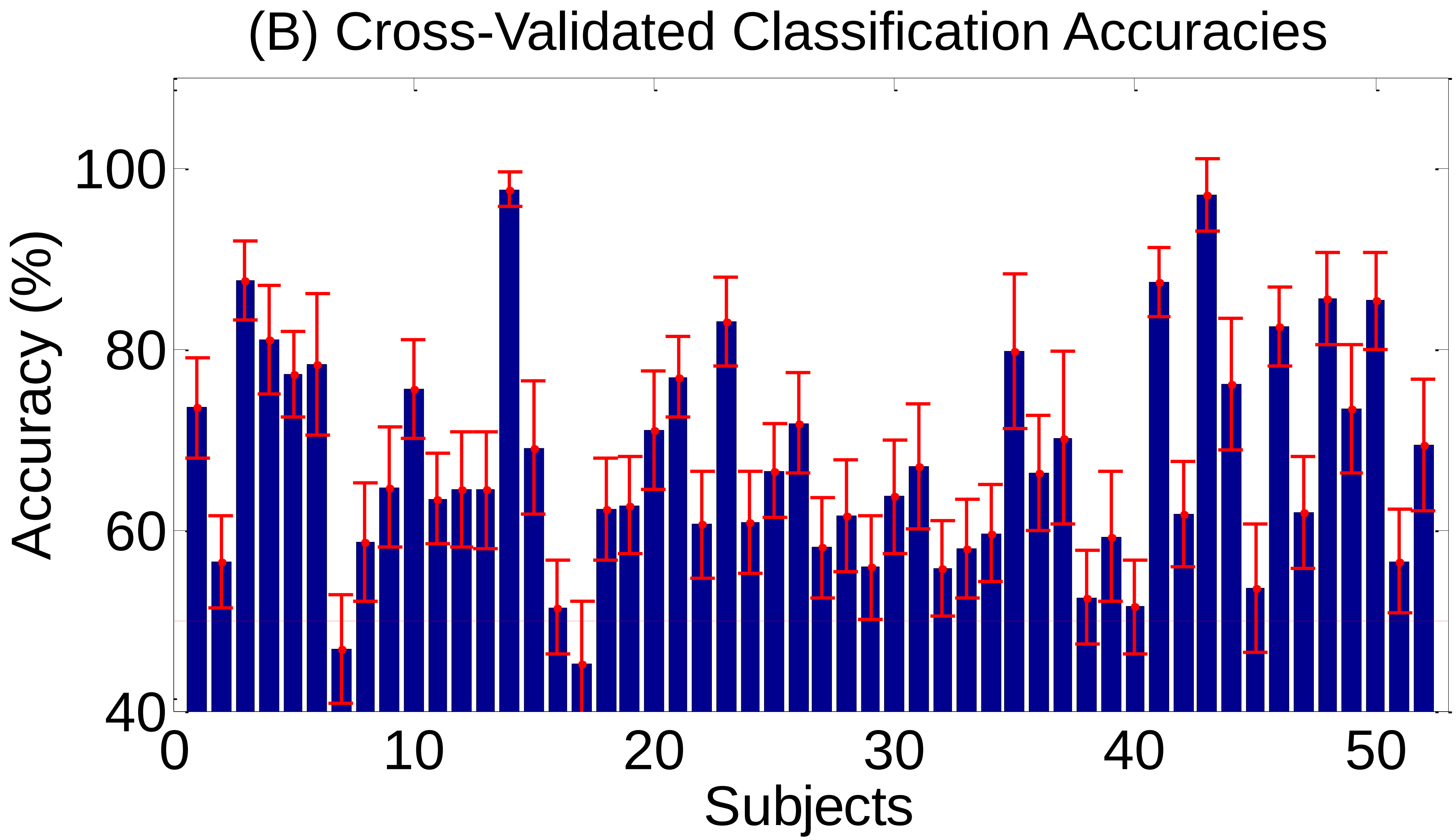

(B) Cross-Validated Classification Accuracies
Accuracy (%)
100
80
60
40
0
10
20
30
40
50
Subjects

Figure 5A

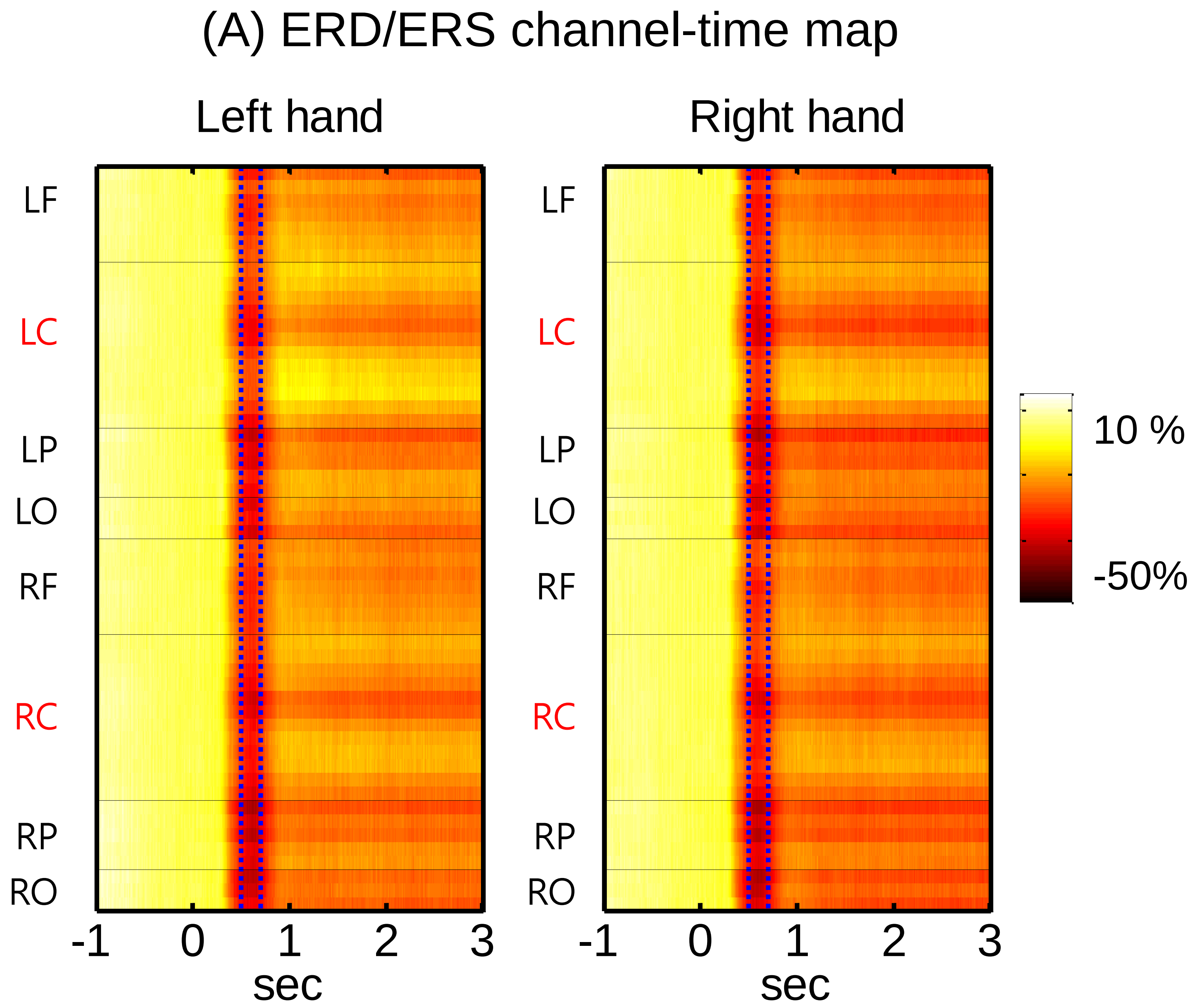
(A) ERD/ERS channel-time map
Left hand
Right hand
LF
LC
LP
LO
RF
RC
RP
RO
10 %
-50%
-1
0
1
2
3
sec

Figure 5B

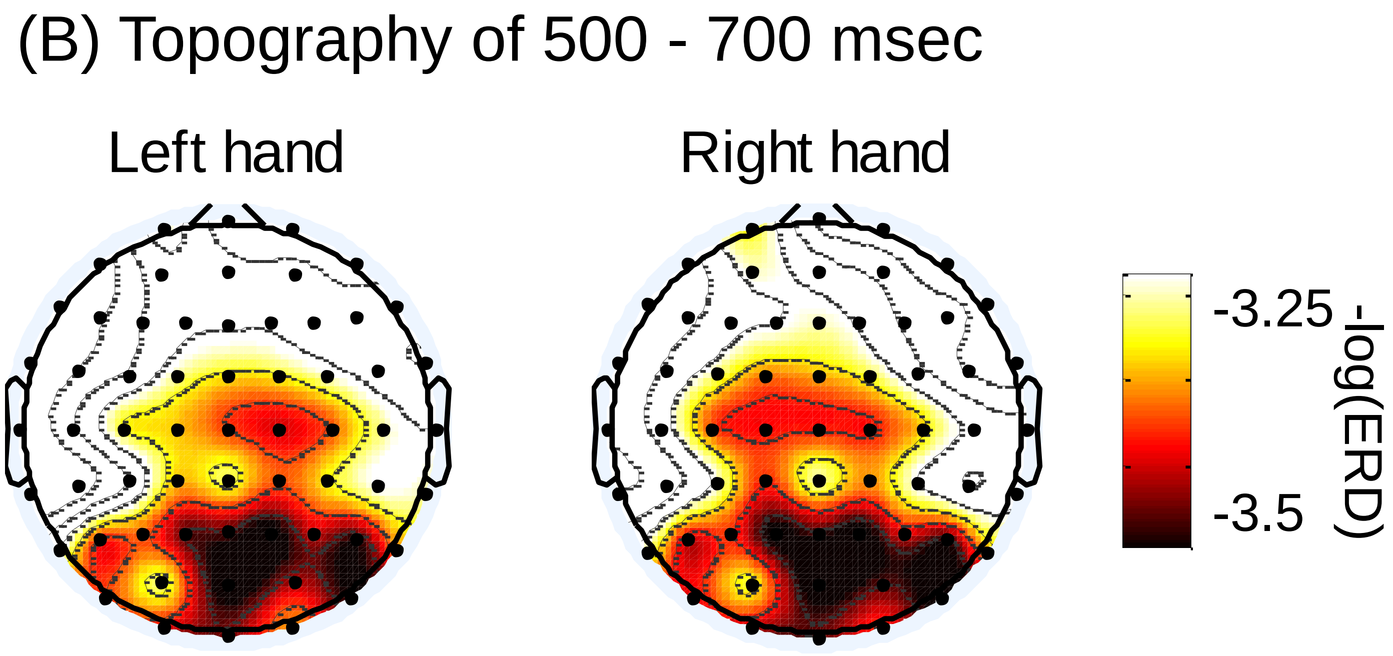
(B) Topography of 500 - 700 msec
Left hand
Right hand
-3.25
-3.5
-log(ERD)

Figure 5C

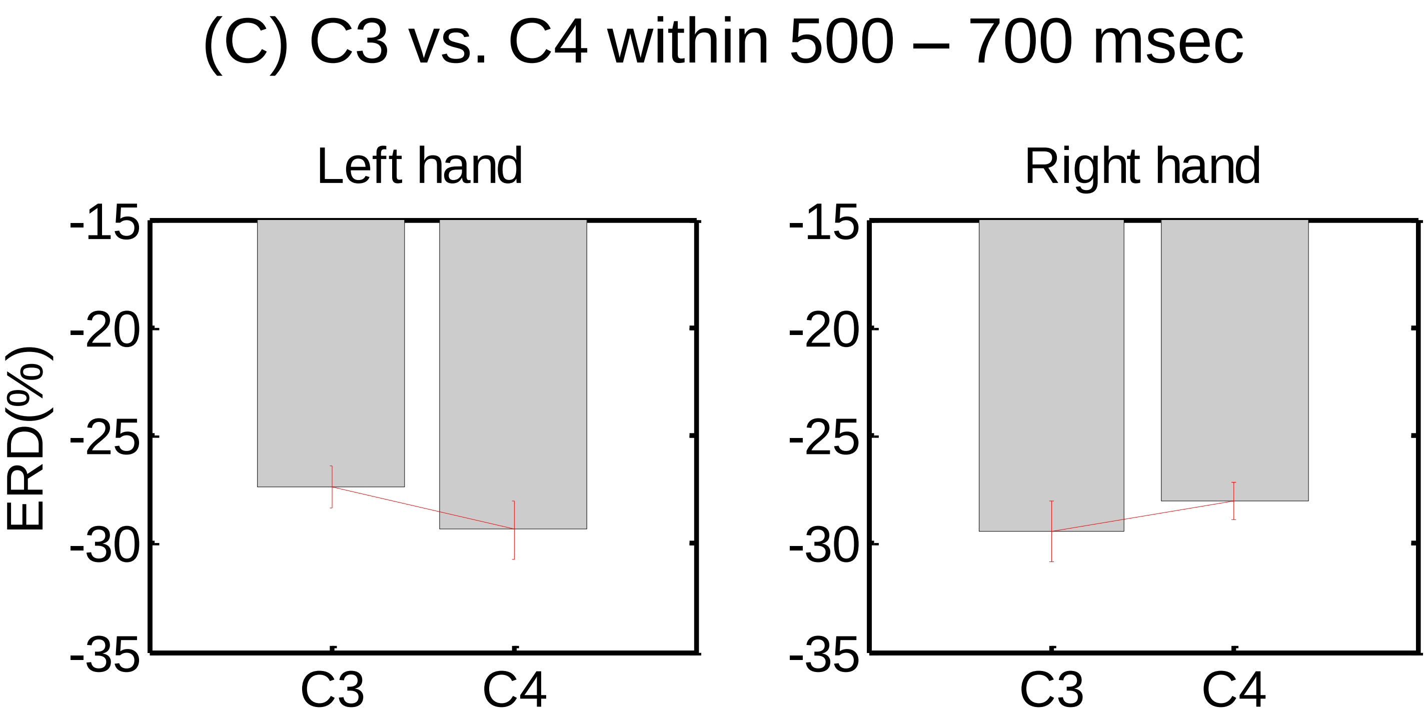
(C) C3 vs. C4 within 500 – 700 msec
Left hand
Right hand
ERD(%)
-15
-20
-25
-30
-35
-15
-20
-25
-30
-35
C3
C4
C3
C4

Figure 6

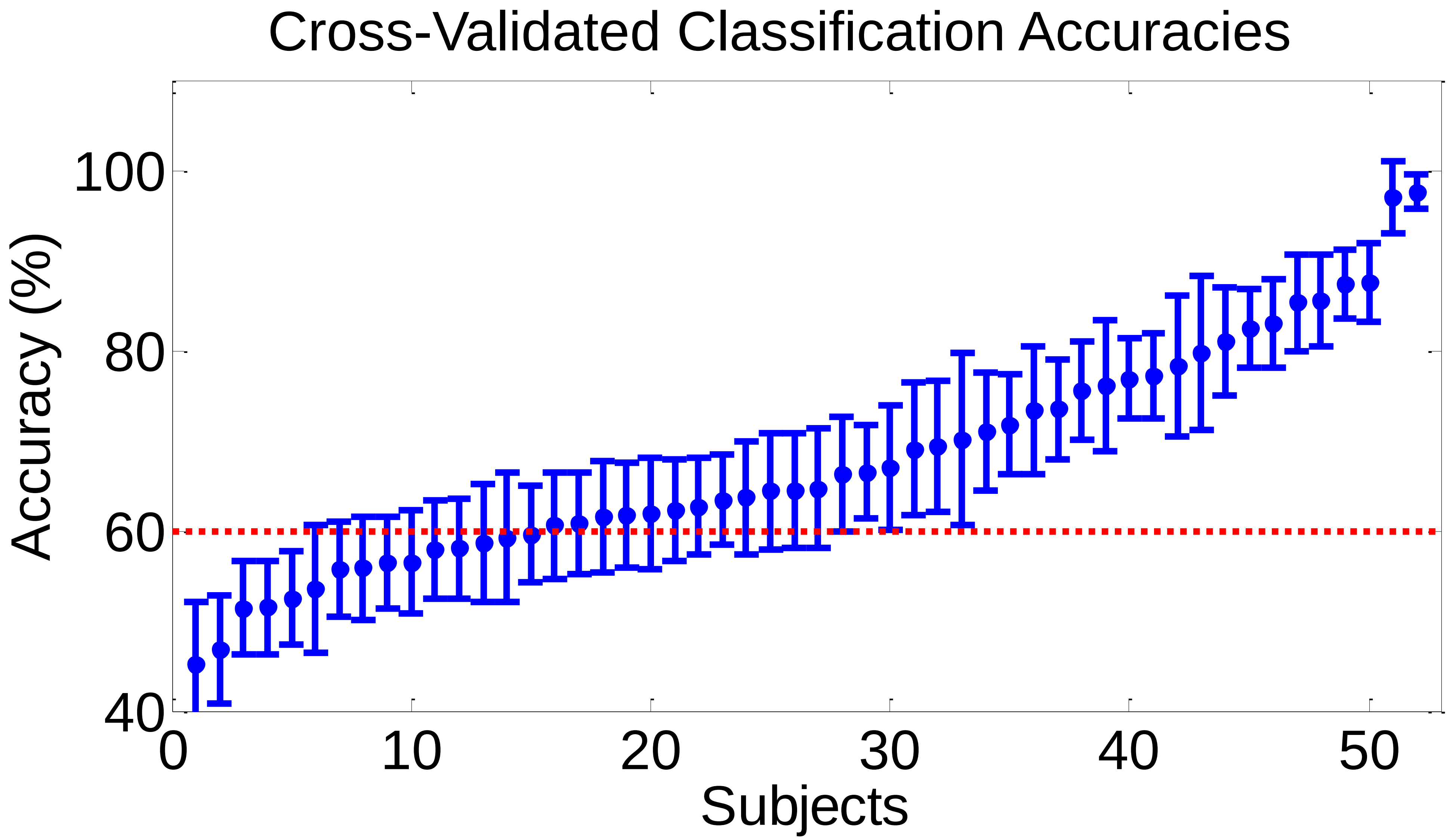
Cross-Validated Classification Accuracies
Accuracy (%)
100
80
60
40
0
10
20
30
40
50
Subjects
