# Supplementary material for: EEG datasets for motor imagery brain–computer interface
Source: Gigascience. 2017 May 4;6(7):1–8. doi: 10.1093/gigascience/gix034 (PMC5493744; doi:10.1093/gigascience/gix034)
Supplement: GIGA-D-16-00104_Revision_1.pdf [file gix034_GIGA-D-16-00104_Revision_1.pdf]

Manuscript

Click here to download Manuscript GigaScience_revised_proof_done_v4.docx

Full title:

EEG datasets for motor imagery brain computer interface

Hohyun **Cho**[1], Minkyu **Ahn**[2], Sangtae **Ahn**[1], Moonyoung **Kwon**[1] and Sung Chan **Jun**[1*]

[1]School of Electrical Engineering and Computer Science, Gwangju Institute of Science and Technology, Gwangju, Korea

[2]Department of Neuroscience, Brown University, Providence, RI, U.S.A. 02912

***** Corresponding author email: scjun@gist.ac.kr (SCJ)

Full address:

Dasan 507, Gwangju Institute of Science and Technology, 123 Cheomdangwagi-ro, Buk-gu, Gwangju 61005, South Korea
TEL: +82-062-715-2216
FAX: +82-062-715-2204

Abstract

Background: Most investigators of brain computer interface (BCI) believe that BCI can be achieved through induced neuronal activity from the cortex, but not by evoked neuronal activity. Motor imagery (MI) based BCI is one of the standard concepts of BCI, in that the user can generate induced activity by imagining motor movements. However, variations in performance over sessions and subjects are too severe to overcome easily; therefore, a basic understanding and investigation of BCI performance variation is necessary to find critical evidence of performance variation.

Here we present not only EEG datasets for MI BCI from 52 subjects, but also the results of a psychological and physiological questionnaire, EMG datasets, the locations of 3D EEG electrodes, and EEGs for non-task related states.

Findings: We validated our EEG datasets by using the percentage of bad trials, event-related desynchronization/synchronization (ERD/ERS) analysis, and classification analysis. After conventional rejection of bad trials, we showed contralateral ERD and ipsilateral ERS in the somatosensory area, which are well-known patterns of MI. Finally, we showed that 63.46% of datasets (33 subjects) included reasonably discriminative information.

Conclusions: Our EEG datasets included good information necessary to determine statistical significance; they consisted of well-discriminated datasets (33 subjects) and less-discriminative datasets. These may provide researchers with opportunities to investigate human factors related to MI BCI performance variation, and may also achieve subject-to-subject transfer by using metadata, including a questionnaire, EEG coordinates, and EEGs for non-task related states.

Keywords

Motor imagery, EEG, brain computer interface, performance variation, subject-to-subject transfer

Data types

EEG datasets

Data size

Data size of one subject: 192 – 226 MB

Total data size of 52 subjects: 9.96 GB

Readme file

Attached as following file name: “README_v1.txt”

Data Description

Background and Purpose

Motor imagery (MI) based brain computer interface (BCI) has attracted great interest recently. Compared with other BCI paradigms, MI BCI can provide users with direct communication without any limb movement or external stimulus (for example, P300 based BCI). MI BCI uses “induced” brain activity [1] from the cortex, rather than “evoked” brain activity. Although the concept of MI BCI is fascinating, it has many obstacles. Among these is the fact that BCI researchers have tended to focus on subject-to-subject transfer (training subject-independent algorithm). To achieve effective subject-to-subject transfer, it is important to understand the variations in performance between subjects [2]. Predicting a subject’s performance by using the resting state, or background noise from EEG, are some examples of this [3–5].

In this paper, we recorded MI BCI EEG and EMG datasets simultaneously with 2 classes (100 or 120 trials for each class) from 52 healthy subjects. We also simultaneously collected 20 trials of real hand movement datasets of EEG and EMG for each subject. To study various forms of evidence of performance variation and subject-to-subject transfer, we collected subjective answers to a psychological and physiological questionnaire, as well as EEG results. In addition, we recorded the locations of 3D EEG electrodes and non-task related EEG (resting state, eyeball and head movements, and jaw clenching). Here, we validated our datasets using the percentage of bad trials, spectral analysis, and classification analysis. These datasets were stored in the GigaScience database, GigaDB.

Experimental Design

Subjects

We conducted a BCI experiment for motor imagery movement (MI movement) of the left and right hands with 52 subjects (19 females, mean age ± SD age = 24.8 ± 3.86); the experiment was approved by the Institutional Review Board of Gwangju Institute of Science and Technology. Each subject took part in the same experiment, and subject ID was denoted and indexed as s1, s2, …, s52. Subjects ‘s20’ and ‘s33’ were both-handed and the other 50 subjects were right-handed. All subjects gave written informed consent to collect information on brain signals and were paid for their participation. The data collected were used only for research purposes.

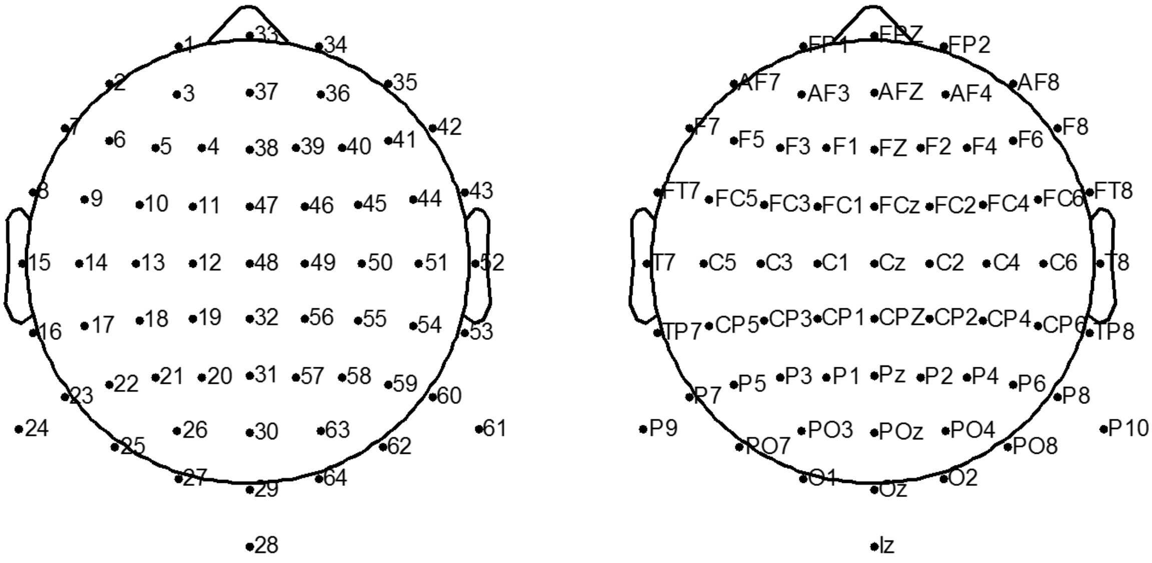

Figure 1. EEG channel configuration–numbering (left) and corresponding labeling (right).

Recording software and device

EEG data were collected using 64 Ag/AgCl active electrodes. As shown in Figure 1, a 64-channel montage based on the international 10-10 system was used to record the EEG signals with 512Hz sampling rates. The EEG device used in this experiment was the Biosemi ActiveTwo system. The BCI2000 system 3.0.2 [6] was used to collect EEG data and present instructions (left hand or right hand MI). Furthermore, we recorded EMG as well as EEG simultaneously with the same system and sampling rate to check actual hand movements. Two EMG electrodes were attached to the flexor digitorum profundus and extensor digitorum on each arm.

For each subject, EEG channel locations (3D coordinates) were collected with a 3D coordinate digitizer (Polhemus Fastrak). Electrode location was measured as the average of three measurements of the digitizer to obtain a stabilized position and prevent hand shaking.

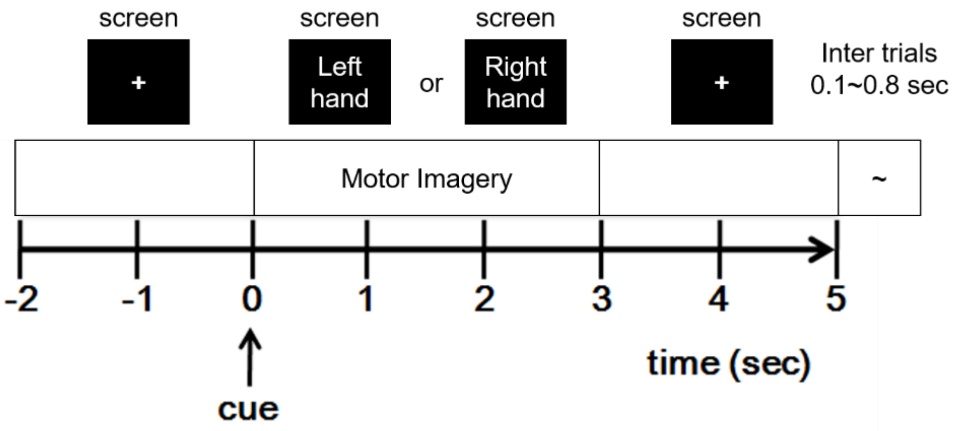

Figure 2. Experimental paradigm. One trial of the MI experiment.

Environment

All experiments were conducted at our laboratory during one of four time slots: T1 (9:30-12:00), T2 (12:30-15:00), T3 (15:30-18:00), or T4 (19:00-21:30). The experiments began on August, 2011 and ended on September, 2011. The background noise level was 37-39 decibels.

Experiment and datasets

For each subject, we recorded data for non-task related and task (MI) related states, as follows:

- **Six types of non-task related data.** We recorded 6 types of noise data (eye blinking, eyeball movement up/down, eyeball movement left/right, head movement, jaw clenching, and resting state) for 52 subjects. Each type of noise was collected twice for 5 seconds, except the resting state, which was recorded for 60 seconds.

- **Real hand movement.** Before beginning the motor imagery experiment, we asked subjects to conduct real hand movements. Subjects sat in a chair with armrests and watched a monitor. At the beginning of each trial, the monitor showed a black screen with a fixation cross for 2 seconds; the subject was then ready to perform hand movements (once the black screen gave a ready sign to the subject). As shown in Figure 2, one of two instructions ("left hand" or "right hand") appeared randomly on the screen for 3 seconds, and subjects were asked to move the appropriate hand depending on the instruction given. After the movement, when the blank screen reappeared, the subject was given a break for a random 4.1 to 4.8 seconds. These processes were repeated 20 times for one class (one run), and one run was performed.

- **MI experiment.** The MI experiment was conducted with the same paradigm as the real hand movement experiment. Subjects were asked to imagine the hand movement depending on the instruction given. Five or six runs were performed during the MI experiment. After each run, we calculated the classification accuracy over one run and gave the subject feedback to increase motivation. Between each run, a maximum 4-minute break was given depending on the subject's demands.

The entire procedure of the experiment is presented in Table 1.

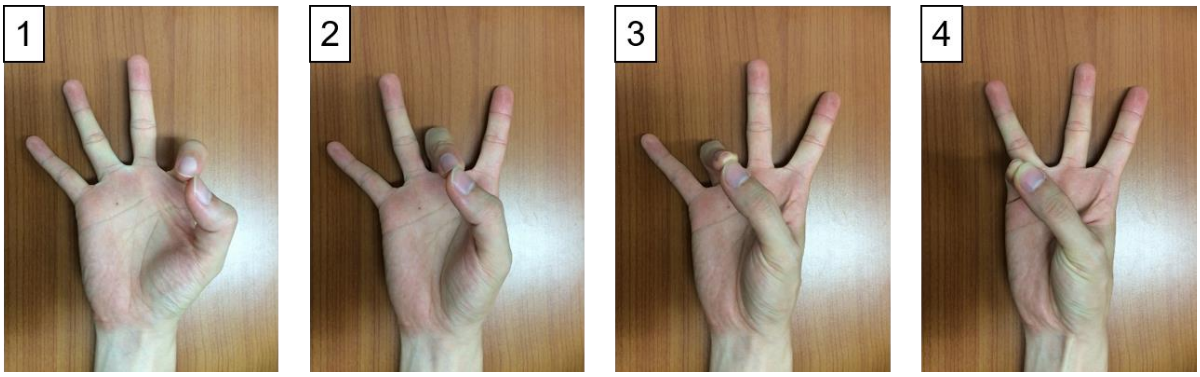

Figure 3. Motor imagery instruction. We asked subjects to imagine four actual finger movements: touching each index, middle, ring, and little finger to the thumb within 3 seconds. Before the MI experiment began, subjects practiced executing the four movements within 3 seconds.

Motor imagery instructions

Before the MI experiment began, we asked each subject to move his/her fingers, starting from the index finger and proceeding to the little finger (depicted in Figure 3) and touching each to their thumb within 3 seconds after onset. Each subject practiced these actual finger movements, and then performed the MI experiment. When imagining the movement, we asked subjects to imagine the kinesthetic experience [7], rather than imagining the visual experience.

Table 1. Experimental procedure

Number	Task	Duration (min)
1	Filling in a consent form and questionnaire	10
2	EEG electrode placement	20
3	Acquisition of the six types of non-task related data	2
4	Practicing actual finger movements	3
5	RUN 1	6
6	Filling out questionnaire	4
7	RUN 2	6
8	Filling out questionnaire	4
9	RUN 3	6
10	Filling out questionnaire	4
11	RUN 4	6
12	Filling out questionnaire	4
13	RUN 5	6
14	Filling out questionnaire	4
15	Online experiment	6
16	Digitizing 3D coordinates of EEG electrodes	15
17	Removing electrodes and cleaning laboratory	20
	Sum	126

Table 2. Questionnaire for motor imagery experiment.

Questionnaire									
Number	**Individual Information**		Subject ID:						
101	Time slot (1 = 9:30/2 = 12:30/3 = 15:30/4 = 19:00)								
102	Handedness (0 = left/1 = right/2 = both)								
103	Age (number)								
104	Sex (female = 0/male = 1)								
105	BCI experience (0 = no/number = how many times)								
106	Biofeedback experience (0 = no/number = how many times)								
	**Before motor imagery experiment**								
107	3. How long did you sleep? (1 = less than 4h/2 = 5~6h/3 = 6~7h/4 = 7~8h/5 = more than 8h)								
108	4. Did you drink coffee within the past 24 hours? (0 = no, number = hours before)								
109	5. Did you drink alcohol within the past 24 hours (0 = no, number = hours before)								
110	6. Did you smoke within the past 24 hours (0 = no, number = hours before)								
111	7. How do you feel?		Relaxed	1	2	3	4	5	Anxious
112			Excited	1	2	3	4	5	Bored
113		Physical state	Very good	1	2	3	4	5	Very bad or tired
114		Mental state	Very good	1	2	3	4	5	Very bad or tired
115	8. BCI performance (accuracy) expected? (%)								
	**During motor imagery experiment**								
	Run 1(after the first Run)								
210	1. Can you continue to the next run? (0 = no/1 = yes)								
211	2. How do you feel?		Relaxed	1	2	3	4	5	Anxious
212			Excited	1	2	3	4	5	Bored
213		Attention level	High	1	2	3	4	5	Low
214		Physical state	Very good	1	2	3	4	5	Very bad or tired
215		Mental state	Very good	1	2	3	4	5	Very bad or tired
216	3. Have you nodded off (slept awhile) during this run? (0 = no/number = how many times)								
217	4. Was it easy to imagine finger movements?		Easy	1	2	3	4	5	Difficult
218	5. How many trials did you miss? (0 = none/number = how many times)								
219	6. BCI performance (accuracy) expected? (%)								
	Run 2 (after the second Run)								
220~229	…								
	Run 3 (after the third Run)								
230~239	…								
	Run 4 (after the fourth Run)								
240~249	…								
	Run 5 (after the fifth Run)								
250~259	…								
	**After the motor imagery experiment**								
301	1. How was this experiment?	Duration	Short	1	2	3	4	5	Long
302		Procedure	Good	1	2	3	4	5	Bad
303		Environment	Comfortable	1	2	3	4	5	Uncomfortable
304	2. BCI performance (accuracy) of whole data expected? (%)								

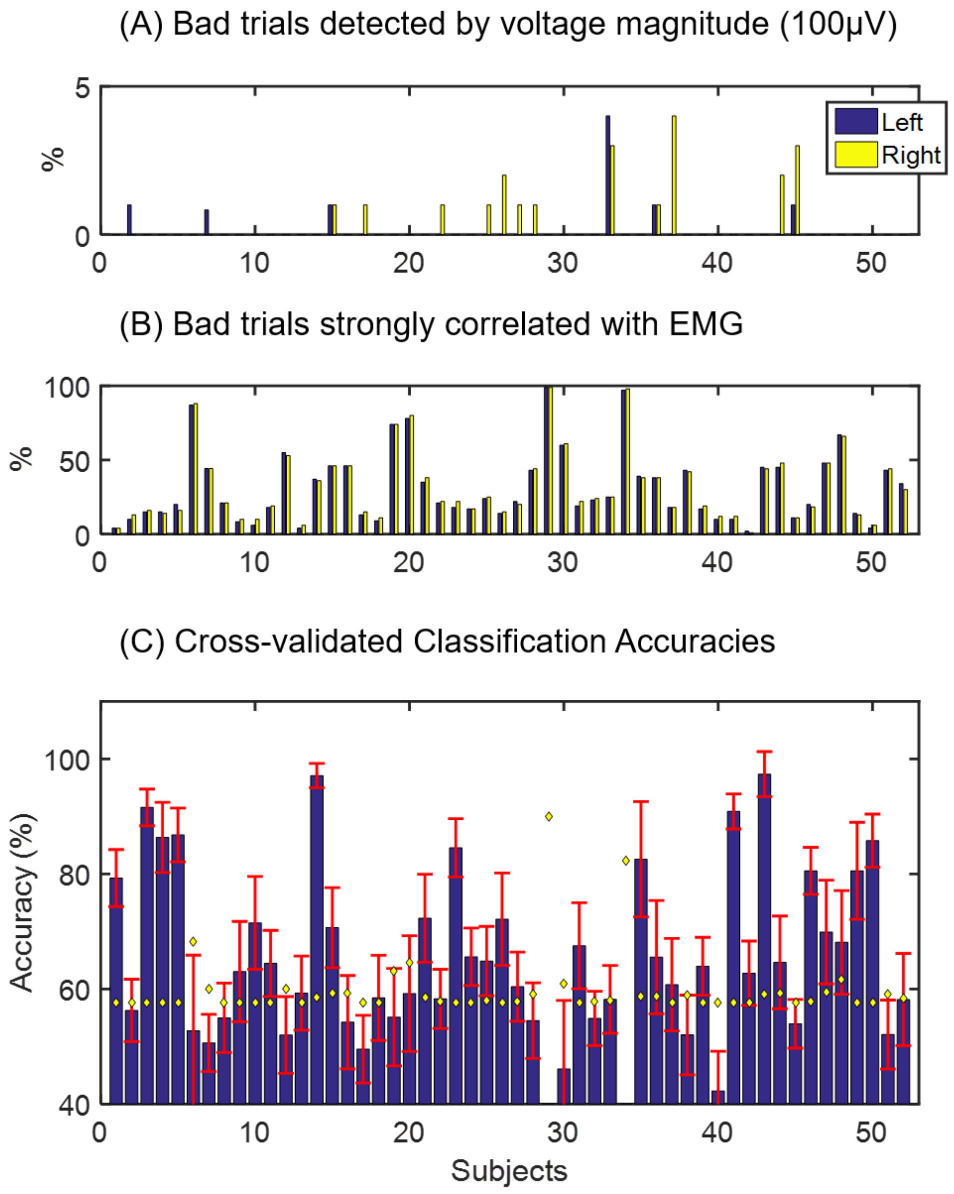

Figure 4. Estimated percentage of bad trials and classification accuracies for all subjects. (A) Each class contained 100 or 120 trials. If any amplitude in 8–30Hz band-passed trial was bigger than 100μV within 500–2500 msec, then the trial was classified as a bad trial. (B) If a power level of 50–250Hz in EMG had correlations with the labels of resting and task-related time points within a motor imagery trial, then it was declared as a bad trial correlated with EMG. (C) Cross-validated classification accuracies were estimated by using Common Spatial Pattern (CSP) and Fisher's linear discriminant analysis (FLDA). We did not include the bad subjects (s29 and s34) severely contaminated by EMG in the classification analysis and the accuracies of these bad subjects were set to zero. Yellow diamonds indicate the random chance levels depending on the number of total trials (excluding bad trials) for each subject.

Questionnaire

We asked subjects to fill out a questionnaire during the MI experiment, as shown in Table 2. Before beginning the MI experiment, subjects answered 15 questions (questions numbered 101 to 115). After every run, subjects answered another 10 questions (questions numbered 210 to

219). After the MI experiment, we asked the subjects to answer a final set of questions (questions numbered 301 to 304). All numerical values of the questions were stored as a Microsoft Excel file (*.xlsx).

Data format and structure

The MATLAB structure of the EEG (1st to 64th channel) and EMG (65th to 68th channel) data ('*.mat') for each subject is shown below:

- rest: resting state with eyes-open condition
- noise:
    - Eyeball movement up/down, 5 sec. × 2
    - Eyeball movement left/right, 5 sec. × 2
    - Jaw clenching, 5 sec. × 2
    - Head movement up/down, 5 sec. × 2
    - Head movement left/right, 5 sec. × 2
- imagery_left: 100 or 120 trials of left hand MI
- imagery_right: 100 or 120 trials of right hand MI
- n_imagery_trials: 100 or 120 trials for each MI class
- imagery_event: value "1" represents onset for each MI trial
- movement_left: 20 trials of real left hand movement
- movement_right: 20 trials of real right hand movement
- n_movement_trials: 20 trials for each real hand movement class
- movement_event: value "1" represents onset for each movement trial
- frame: temporal range of a trial in milliseconds
- srate: sampling rate
- senloc: 3D sensor locations
- psenloc: sensor location projected to unit sphere
- subject: subject's two-digit ID - 's#'
- comment: comments for the subject
- bad_trial_indices
    - bad trials determined by voltage magnitude

- bad trials correlated with EMG activity

Reliability

Methods

For preprocessing, we used Butterworth filtering with 4th order for high-pass and band-pass filtering. We validated the EEG datasets in three different ways:

- First, we checked the number of bad trials in each subject's data. If a band-passed (8–30 Hz) trial had an amplitude greater than ±100 µV [8–10] within 500–2500 msec, the trial was declared bad. The frequency band is involved in somatosensory rhythm (SMR) [1,11,12]. The time window was determined by an algorithm for selection of a discriminative time interval (see Appendix in [12]). The percentage of bad trials was estimated for each subject. The bad trials were not considered in the following analysis. The bad trial indexes were added for each subject dataset, as shown in the section titled 'Data format and structure.'

- Secondly, we investigated whether each trial is correlated with EMG (e.g., real hand movement) adopting [13]'s idea which was using correlation between class labels and EMG activity. In the prescreening of EMG in the real hand movement experiment, we observed high frequency activity (50–250 Hz) during real hand movement. We calculated Spearman's correlation (non-parametric)—which is less sensitive to outliers in the data—between the EMG power of high frequency activity and the label of time points, as follows:

  1. High-pass filtering of all EMG trials above 0.5 Hz to remove drifts;
  2. Common average reference;
  3. Band-pass filtering of all trials with 50–250 Hz;
  4. Hilbert transform;
  5. Take absolute and squared magnitudes for each complex value of all trials;
  6. Extract data in resting window (-1000–0 msec) and task-related window (0–3000 msec) for each trial;
  7. Prepare labels for each time point within a trial:
     - Tag '-1' value for time points in resting window;
     - Tag '+1' value for time points in task-related window;
  8. Calculate Spearman's correlation between squared magnitudes and label of time points;
  9. Execute permutation test over time points within a trial:
     - Calculate Spearman's correlation between permuted features and labels;
     - Repeat 100 times;

- Make probability density function (PDF) of the values of Spearman's correlation;
- Calculate p-values (one right tailed test) over all trials and four EMG channels;

10. If false discovery rate (FDR) corrected p-value is smaller than 0.01, then it is declared as a bad trial correlated with EMG.

Finally, the EMG correlated EEG trial indices were added for each subject dataset, as shown in the 'Data format and structure' section.

- Thirdly, we checked event-related desynchronization/synchronization (ERD/ERS) of SMR for each subject [1]. To calculate ERD/ERS for each channel, we followed the same procedure as that in [1], as follows:

1. High-pass filtering of all EEG trials above 0.5 Hz to remove drifts;
2. Common average reference;
3. Band-pass filtering of all trials with 8–14 Hz;
4. Hilbert transform of all trials;
5. Absolute magnitude taken for each complex value of all trials;
6. Magnitude of Hilbert transformed samples averaged across all trials;
7. Baseline correction for each trial to obtain a percentage value for ERD/ERS per the formula $\mathrm{ERD\%} = \frac{\mathrm{A-R}}{\mathrm{R}} \times 100$, where A is each time sample and R is the mean value of the baseline period (-500 to 0 msec).

- Lastly, we validated the discriminability of the left versus right hand MI EEG data as classification accuracy. All trials for each subject were pre-processed by high-pass filtering and common average reference, and then filtered both spectrally (8–30Hz) and temporally (0.5–2.5 seconds after stimulus onset). For the feature extraction algorithm, we used 2 spatial filters of the common spatial pattern (CSP) for each class [11,12]. For classification, we used Fisher's linear discriminant analysis (FLDA). We performed cross-validation in the following way. For each class, we divided all trials of MI data into 10 subsets each. Seven subsets were chosen randomly and used to train CSP and FLDA, and the remaining 3 subsets were used to test them. This procedure was repeated 120 times by choosing 3 among the 10 subsets randomly. Finally, 120 classification accuracies were estimated and averaged.

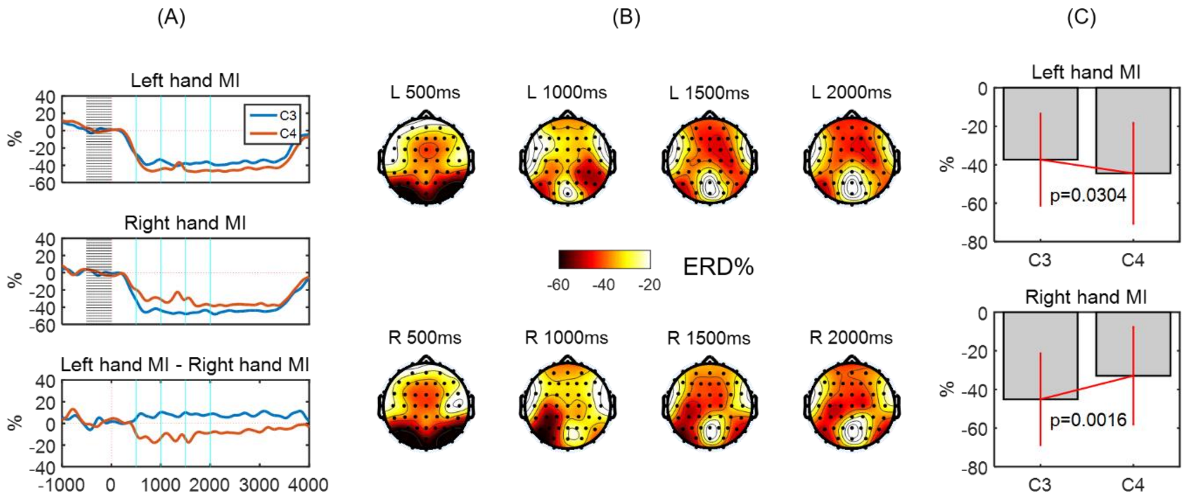

Figure 5. Event-related desynchronization (ERD) of somatosensory rhythm (8–14Hz). (A) The first and second rows show ERD of the C3 and C4 channels in left and right hand motor imagery, respectively. The last row shows the difference in ERDs between left and right motor imagery. The gray shaded region is the baseline period. Cyan colored vertical lines represent time points such as 500, 1000, 1500, and 2000 msec. (B) Topographies of ERDs at the cyan colored time points in (A). Initials "L" and "R" indicate left and right motor imagery movements, respectively. (C) Comparison of ERD at C3 and C4 channels within 500–2500 msec. P-values were estimated by paired t-test.

Results

Percentage of bad trials. We calculated the percentage of bad trials for each subject, as shown in Figure 4A. The percentages of bad trials within the spectral and temporal discriminative ranges were below 5% for most subjects. Furthermore, we calculated the percentage of EMG trials correlated with labels of time points for each MI trial, as shown in Figure 4B. Two subjects (s29 and s34) showed that more than 90% of their trials were correlated with EMG; most of the trials demonstrated a greater power of high frequency EMG (50–250 Hz) in the task-related period after onset than the resting period before onset. Thus, these two subjects were declared as bad subjects and were discarded in the further analysis. Rest of subjects has at least 10 trials per class. The literature [8] showed that the upper confidence limits of chance with α = 5% were 70% (classification accuracy) in a 2-class problem with 10 trials for each class. If a subject has higher accuracy than the random chance level depending on the number of trials, we classified the subjects into discriminative group. On the other hand, we applied the same method to real hand movement trials to test our method. Most of the trials were correlated to the power of high frequency EMG. Here, although we set the p-value threshold as 0.05, a few trials were not correlated with the labels of resting or task-related states. Thus, our threshold was 0.01; we applied the same threshold to MI data.

Most existing studies detected EMG activity through manual monitoring. They recorded EMG and EEG simultaneously and monitored EMG burst during the experiment. On the other hand, in a literature [14], the resting state of EMG was recorded and the significant threshold from the resting EMG was defined. Furthermore, in another literature [13], correlation values between target position (cursor movement control application in BCI) and EMG activity were calculated, and they were compared with the correlation values of EMG-class labels and EEG-

class labels. Also, according to the literature [14], t-values between the EMGs of the operant hand and the non-operant hand were calculated. We believed that the correlation between EMG activity and time point labels within a trial could provide the solution for detecting which EEG trial was correlated to EMG. We attempted the voltage thresholding method, but there were trials correlated with EMG even EMG activity was smaller than threshold. We also tried to compare the voltage distribution between the resting state and task-related EMG, but there were trials correlated to EMG although the distribution of EMG of a trial has a distribution similar to that of the resting state. Finally, Thirty-three subjects had higher classification accuracy than their own random chance (yellow diamond marker) with a confidence level of α = 5%, as shown in Figure 4C.

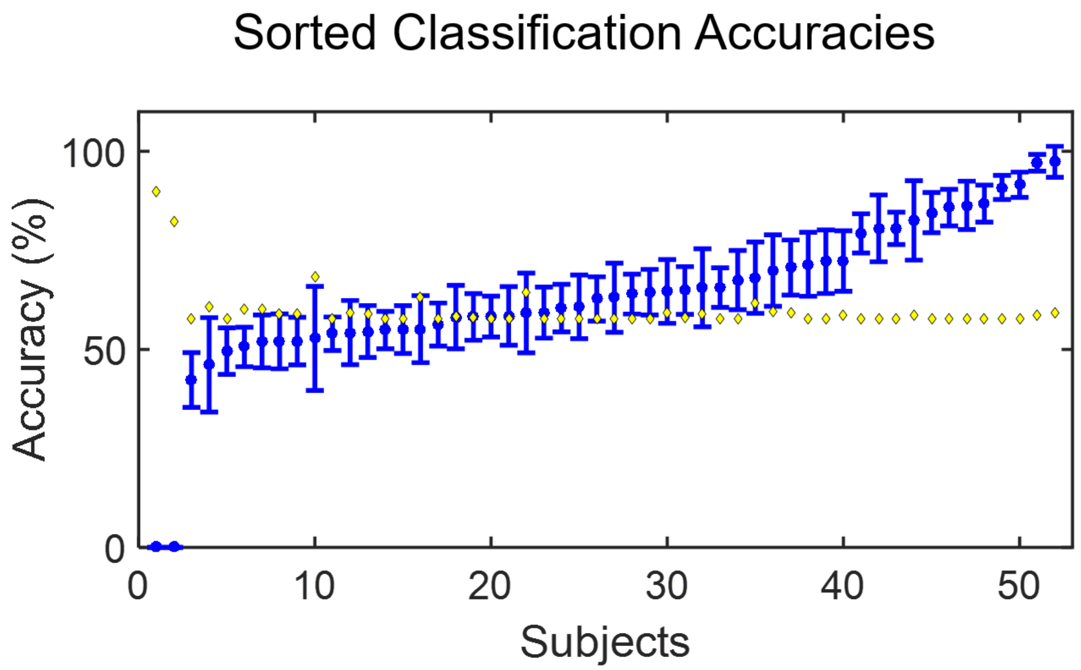

Figure 6. Sorted cross-validated classification results. Sorted accuracies are depicted in increasing order. Nineteen subjects showed low BCI performance (< random chance marked with yellow diamond). Because of sorting, the number on the X-axis does not correspond to subject numbers “s01” to “s50.”

ERD/ERS. The ERD/ERS results of mu rhythm (8–14 Hz) are depicted in Figure 5. Figure 5A shows the grand averaged ERD/ERS (%) of C3 and C4 channel over the 33 subjects who has discriminative information (as shown in Figure 4C). The powers of mu rhythm in C3 and C4 channel decreased in both left and right hand MI. Contralateral channel showed bigger desynchronization in corresponding class. The last row in Figure 5A shows the difference over time of ERD/ERS in C3 and C4 channels. The C4 channel showed a bigger difference than the C3 channel. Figure 5B shows the topographies at specific time points, for instance, 500, 1000, 1500, and 2000 msec. Those time points are marked in Figure 5A as a cyan colored vertical line. At 500 msec, the fronto-central and occipital areas were activated (‘alpha inhibition [1,15]’). The activation of the occipital area may be related to processing of visual stimulus. The fronto-central area may be related to the frontal motor area for planning motor imagery [16,17]. At 1000 msec, contralateral channels showed bigger ERD than ipsilateral channels. For left hand MI, the right central and parietal areas showed bigger ERD than the left hemisphere. At 1500 msec, the frontal area was activated. We expect that the activation of the

frontal area is related to control or modulation of other sensorimotor areas [16,17]. In Figure 5C, bar graphs of left hand MI show that the contralateral ERD (C4 channel) is stronger than the ipsilateral ERD (C3 channel).

Classification. The mean accuracy of all BCI performances (Figure 4C) over the 50 subjects, excluding bad subjects, was 63.54% (±18.65%) in our datasets. In BCI2000 MI datasets [6,18,19], the average accuracy was 60.42% (±11.68%) over 109 subjects using CSP and FLDA [6,18–21]. In our datasets, nineteen subjects (36.54% of 52 subjects) showed low BCI performance (below random chance, which is the upper confidence limit of chance with α = 5%), as shown in Figure 6. This is greater than a report on 99 subjects [22] that showed that 6.7% of the subjects had accuracies lower than 60% (here, the average accuracy over the 99 subjects was not reported). Comparing with the datasets of [20], our datasets have more trials, even though bad trials were rejected and excluded from results. [20] has MI data of 109 subjects, but the number of total trials for each subject is about 20 trials, which has a random chance level of 65% (α=5%).

Availability of supporting data

Supporting data in this paper can be found in the GigaScience database, GigaDB.

Abbreviations

BCI: Brain Computer Interface; EEG: Electroencephalography; ERD/ERS: Event-related desynchronization/synchronization; SMR: Somatosensory Rhythm; MI: motor imagery

Competing interests

The authors declare that they have no competing interests.

Authors' contributions

MA and HC designed the experiments. HC, SA, MA and MK performed the experiment. HC and MA designed data the validation method and analyzed the data. HC prepared datasets for storing. SCJ and HC wrote the paper. All authors read and approved the final manuscript.

Acknowledgements

This work was supported by the GRI (GIST Research Institute) in 2016 and the National Research Foundation of Korea (NRF) funded by the Ministry of Science, ICT & Future Planning (NRF-2016M3C7A1905475, NRF-2013R1A1A2009029).
.

References

1. Pfurtscheller G, Lopes da Silva FH. Event-related EEG/MEG synchronization and desynchronization: basic principles. Clin. Neurophysiol. 1999;110:1842–57.

2. Grosse-Wentrup M, Schölkopf B. A review of performance variations in SMR-based brain-computer interfaces (BCIs). Brain-Comput. Interface Res. [Internet]. Springer; 2013 [cited 2016 Dec 21]. p. 39–51. Available from: http://link.springer.com/chapter/10.1007/978-3-642-36083-1_5

3. Blankertz B, Sannelli C, Halder S, Hammer EM, Kübler A, Müller K-R, et al. Neurophysiological predictor of SMR-based BCI performance. NeuroImage. 2010;51:1303–9.

4. Ahn M, Cho H, Ahn S, Jun SC. High Theta and Low Alpha Powers May Be Indicative of BCI-Illiteracy in Motor Imagery. PLoS ONE. 2013;8:e80886.

5. Cho H, Ahn M, Kim K, Jun SC. Increasing session-to-session transfer in a brain–computer interface with on-site background noise acquisition. J. Neural Eng. 2015;12:66009.

6. Schalk G, McFarland DJ, Hinterberger T, Birbaumer N, Wolpaw JR. BCI2000: a general-purpose brain-computer interface (BCI) system. IEEE Trans. Biomed. Eng. 2004;51:1034–43.

7. Neuper C, Scherer R, Reiner M, Pfurtscheller G. Imagery of motor actions: Differential effects of kinesthetic and visual–motor mode of imagery in single-trial EEG. Cogn. Brain Res. 2005;25:668–677.

8. Müller-Putz G, Scherer R, Brunner C, Leeb R, Pfurtscheller G. Better than random: A closer look on BCI results. Int. J. Bioelectromagn. 2008;10:52–55.

9. Muthukumaraswamy S. High-frequency brain activity and muscle artifacts in MEG/EEG: a review and recommendations. Front. Hum. Neurosci. 2013;7:138.

10. van Dinteren R, Arns M, Jongsma ML, Kessels RP. P300 development across the lifespan: a systematic review and meta-analysis. PLoS One. 2014;9:e87347.

11. Ramoser H, Muller-Gerking J, Pfurtscheller G. Optimal spatial filtering of single trial EEG during imagined hand movement. IEEE Trans. Rehabil. Eng. 2000;8:441–6.

12. Blankertz B, Tomioka R, Lemm S, Kawanabe M, Muller K-R. Optimizing Spatial filters for Robust EEG Single-Trial Analysis. IEEE Signal Process. Mag. 2008;25:41–56.

13. Vaughan TM, Miner LA, McFarland DJ, Wolpaw JR. EEG-based communication: analysis of concurrent EMG activity. Electroencephalogr. Clin. Neurophysiol. 1998;107:428–433.

14. Matsumoto J, Fujiwara T, Takahashi O, Liu M, Kimura A, Ushiba J. Modulation of mu rhythm desynchronization during motor imagery by transcranial direct current stimulation. J. Neuroengineering Rehabil. 2010;7:1.

15. Jensen O, Mazaheri A. Shaping functional architecture by oscillatory alpha activity: gating by inhibition. Front. Hum. Neurosci. 2010;4:186.

16. Hanakawa T, Immisch I, Toma K, Dimyan MA, Gelderen PV, Hallett M. Functional Properties of Brain Areas Associated With Motor Execution and Imagery. J. Neurophysiol. 2003;89:989–1002.

17. Solodkin A, Hlustik P, Chen EE, Small SL. Fine Modulation in Network Activation during Motor Execution and Motor Imagery. Cereb. Cortex. 2004;14:1246–55.

18. Goldberger AL, Amaral LA, Glass L, Hausdorff JM, Ivanov PC, Mark RG, et al. Physiobank, physiotoolkit, and physionet components of a new research resource for complex physiologic signals. Circulation. 2000;101:e215–e220.

19. BCI2000 wiki [Internet]. Available from: www.bci2000.org

20. EEG Motor Movement/Imagery Dataset [Internet]. [cited 2016 Dec 20]. Available from: https://physionet.org/pn4/eegmmidb/

21. Cho H, Ahn S, Jun SC. How is subject-to-subject transfer probable in motor imager BCI? Proceeding Sixth Int. Brain-Comput. Interface Meet. 2016;Article ID: 167.

22. Guger C, Edlinger G, Harkam W, Niedermayer I, Pfurtscheller G. How many people are able to operate an EEG-based brain-computer interface (BCI)? IEEE Trans. Neural Syst. Rehabil. Eng. Publ. IEEE Eng. Med. Biol. Soc. 2003;11:145–7.

Figure 1

Click here to download Figure figures_v4.pptx

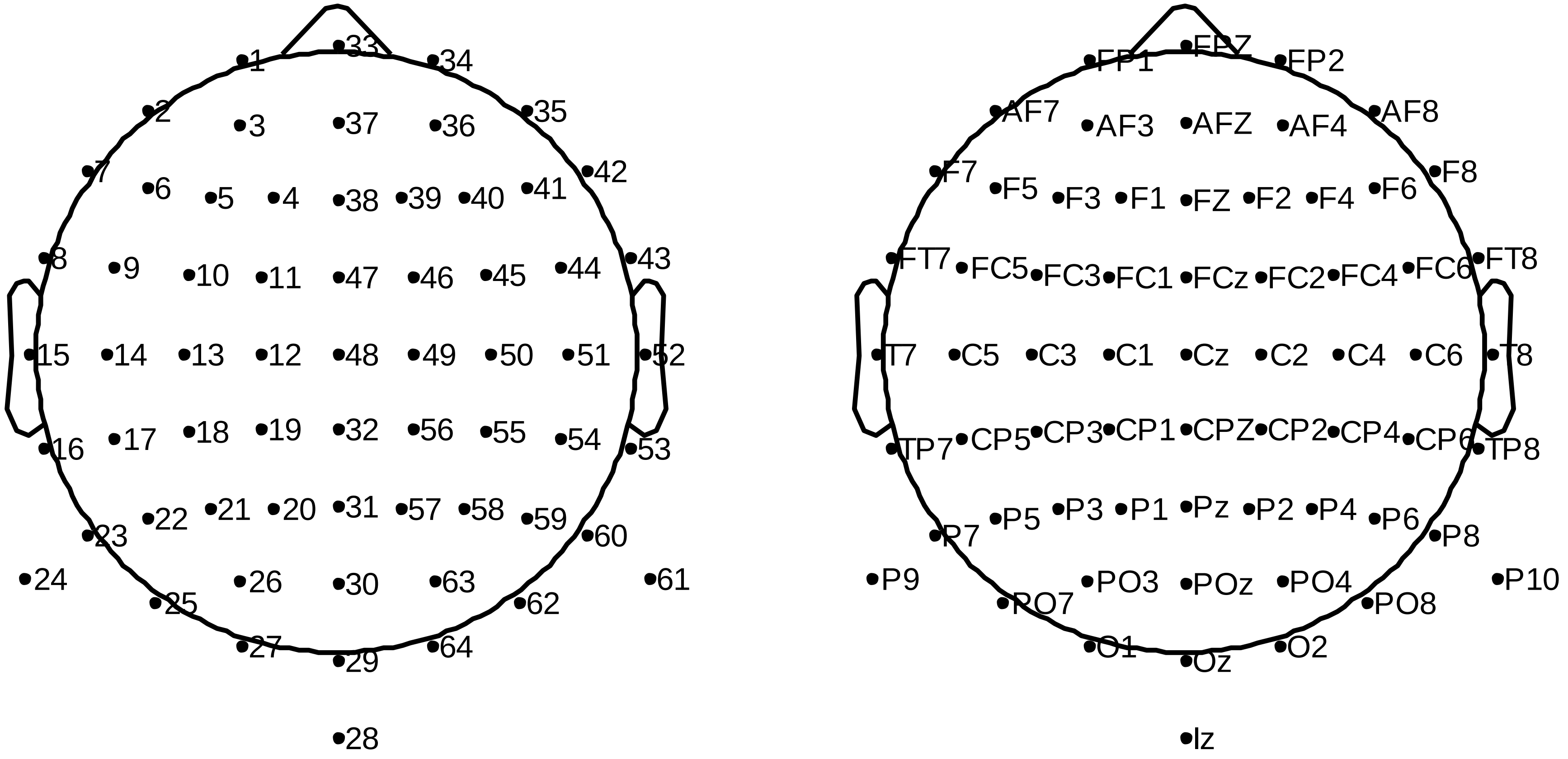

Figure 2

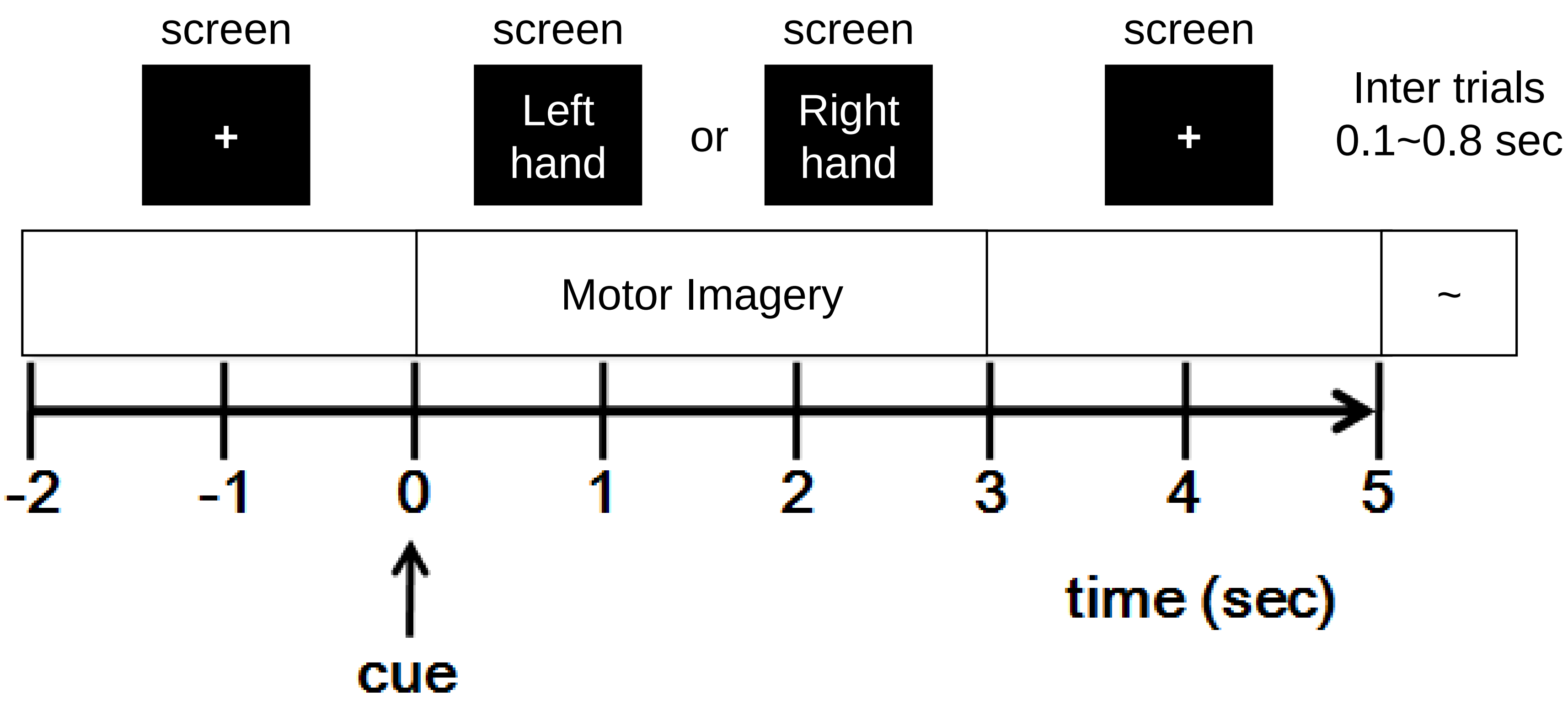
screen
+
screen
Left hand
or
screen
Right hand
screen
+
Inter trials 0.1~0.8 sec
Motor Imagery
~
-2
-1
0
1
2
3
4
5
cue
time (sec)

Figure 3

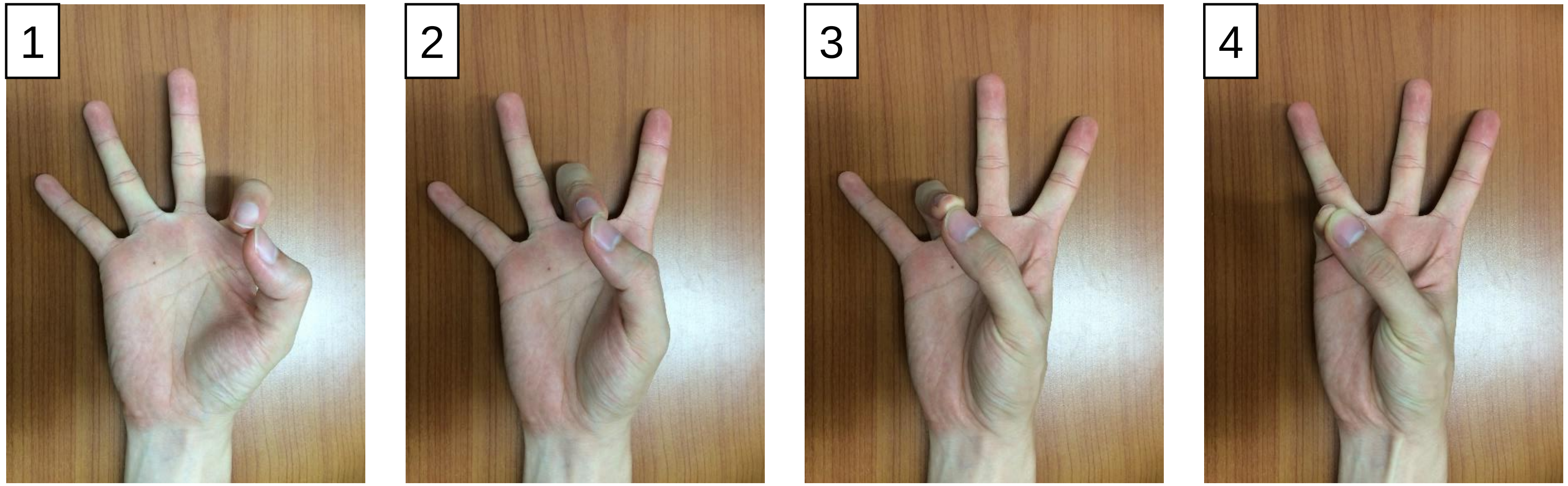

Figure 4A

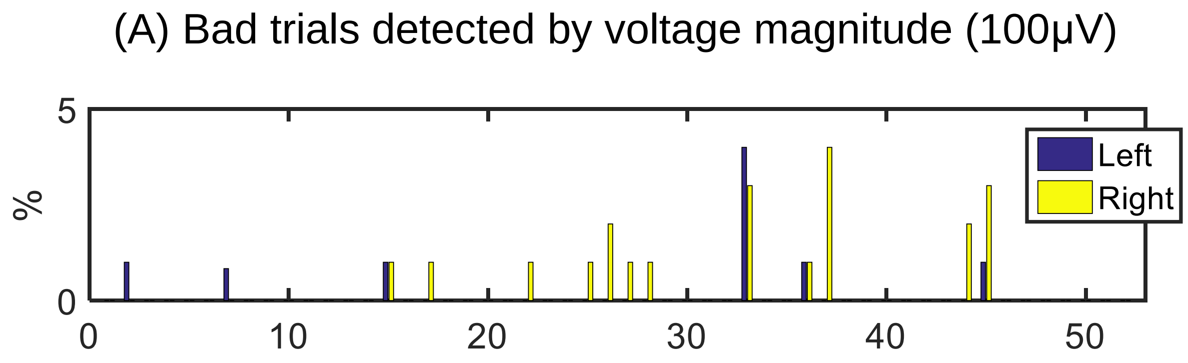
(A) Bad trials detected by voltage magnitude (100μV)
5
%
0
0
10
20
30
40
50
Left
Right

Figure 4

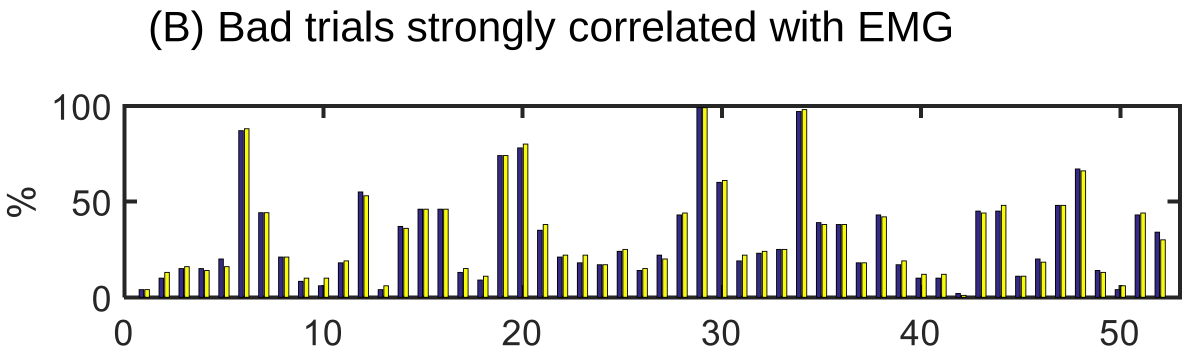

(B) Bad trials strongly correlated with EMG
%
100
50
0
0
10
20
30
40
50

Figure 4

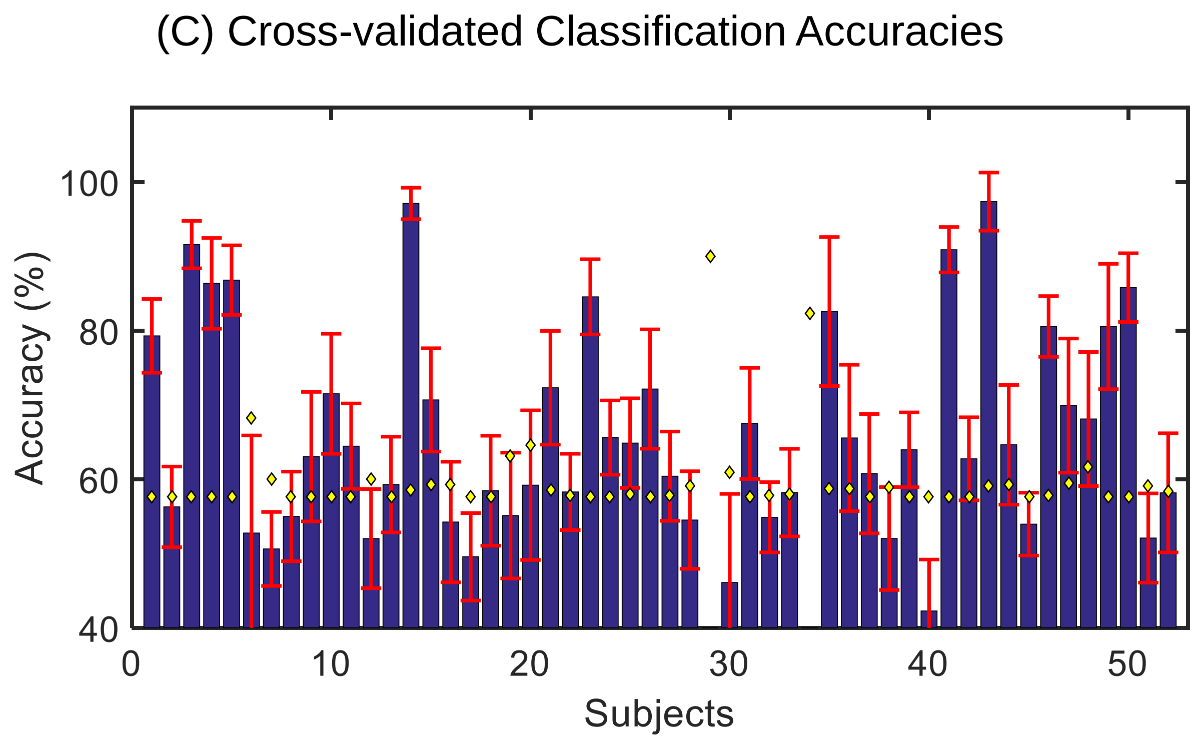
(C) Cross-validated Classification Accuracies
Accuracy (%)
Subjects
100
80
60
40
0
10
20
30
40
50

Figure 5A

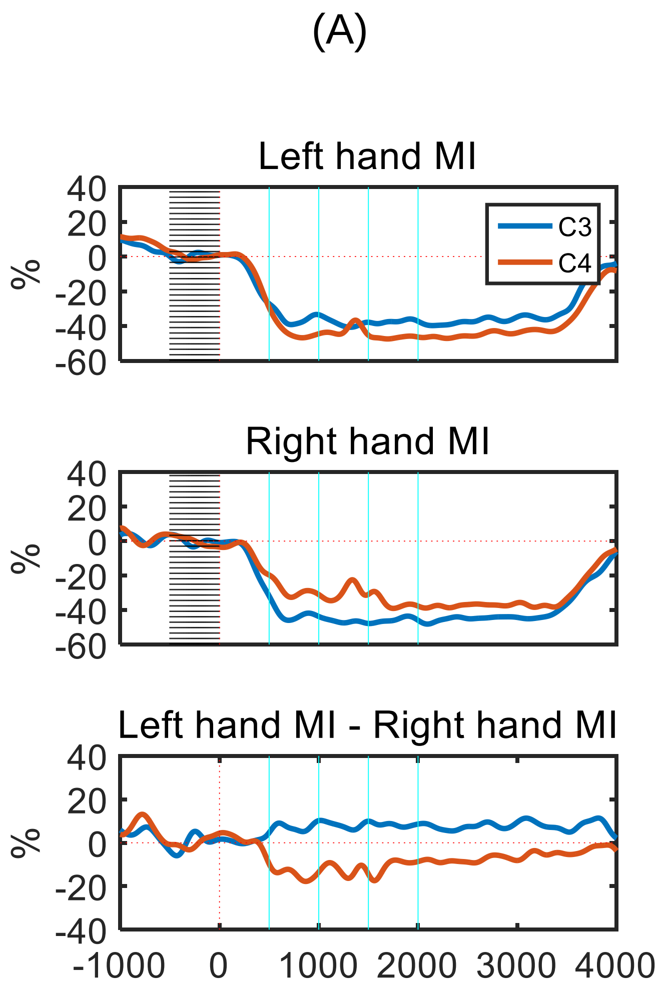

(A)
Left hand MI
C3
C4
%
40
20
0
-20
-40
-60
Right hand MI
Left hand MI - Right hand MI
-1000
0
1000
2000
3000
4000

Figure 5

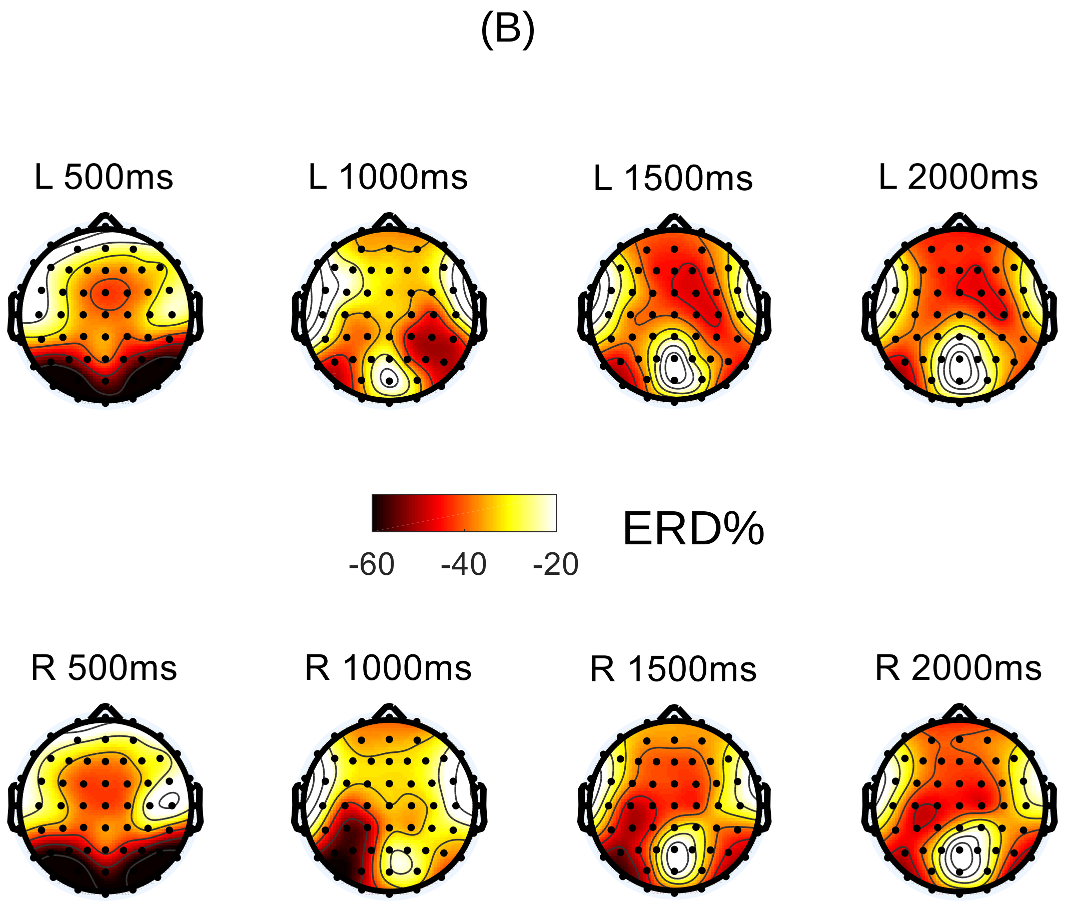
(B)
L 500ms
L 1000ms
L 1500ms
L 2000ms
-60
-40
-20
ERD%
R 500ms
R 1000ms
R 1500ms
R 2000ms

Figure 5

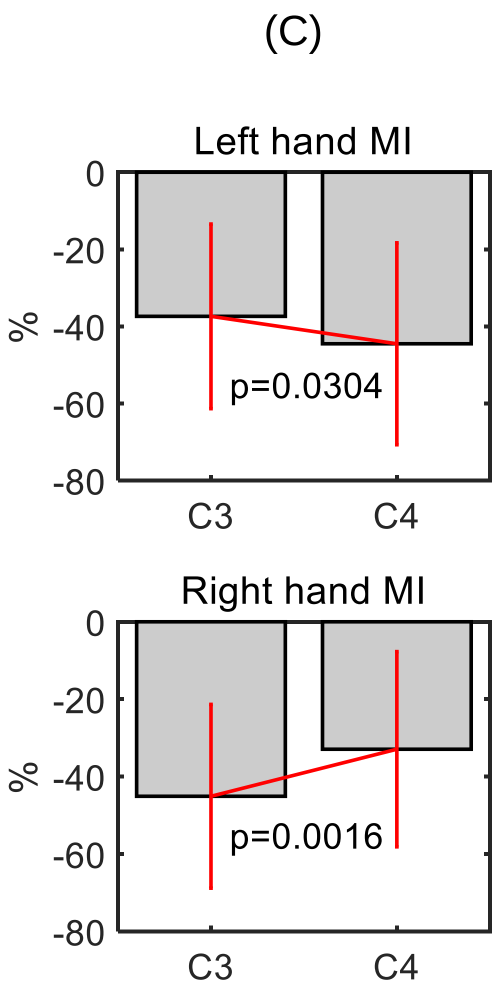
(C)
Left hand MI
0
-20
-40
-60
-80
%
p=0.0304
C3
C4
Right hand MI
0
-20
-40
-60
-80
%
p=0.0016
C3
C4

Figure 7

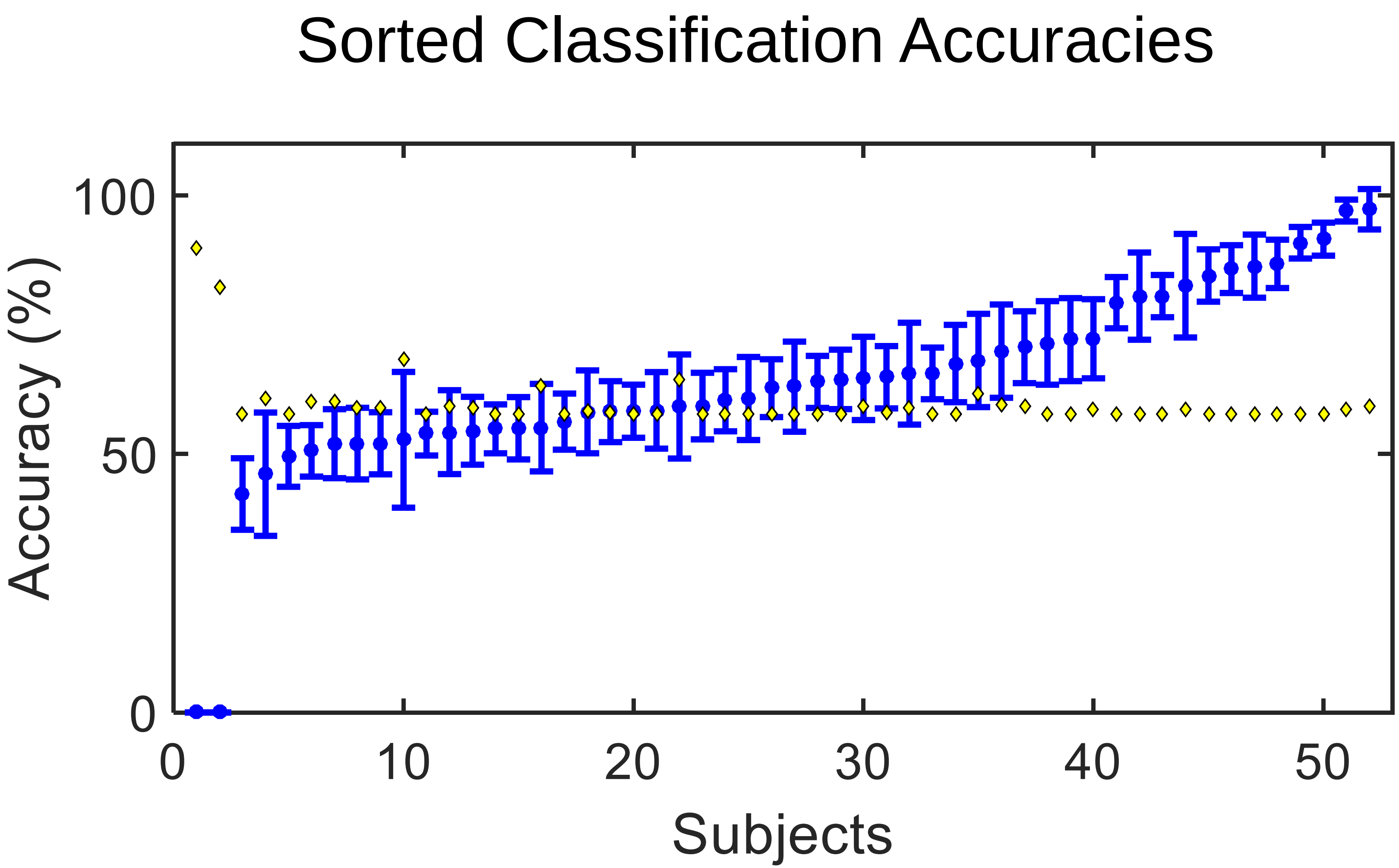

Sorted Classification Accuracies
100
50
0
0
10
20
30
40
50
Accuracy (%)
Subjects

Click here to access/download
Supplementary Material
response-to-reviewers-v4.pdf
