# Supplementary material for: EEG datasets for motor imagery brain–computer interface
Source: Gigascience. 2017 May 4;6(7):1–8. doi: 10.1093/gigascience/gix034 (PMC5493744; doi:10.1093/gigascience/gix034)
Supplement: Response_to_reveiwer_comments_Original_Submission.pdf [file gix034_Response_to_reveiwer_comments_Original_Submission.pdf]

More readable Response-to-Reviewers letter was added as 'Supplementary Material'. Please check it as you wish.

Reviewer 1

COMMENTS TO THE AUTHOR(S)

Q1) This manuscript describes an EEG motor imagery (MI) data set collected from 52 subjects. The analysis is primarily to confirm the validity of the data and is not novel. Such consistently-collected and comparatively large data sets are of interest to EEG-based brain-computer interface (BCI) researchers. Another similar and large EEG MI data set is available on PhysioNet, but additional large and consistent data sets are beneficial to the research community. The present data set is also unique in that it includes other non-EEG measurements, such as EMG and EOG, and subjective evaluations that can be useful for a variety of analyses.
There are a few items that should be addressed in order to improve the utility of this data set. For MI data, it is desirable to have EEG from actual movements as a reference of the spatio-spectral characteristics of the EEG during movement for each subject. Without this, it is difficult to determine if the MI non-responders weren't performing the imagery properly, do not have prominent sensorimotor rhythms, etc. Since EMG and other non-EEG measurements have been acquired, for the data validation, it is desirable to validate or screen for trials containing EMG that was correlated to the MI. Otherwise, some part of the EEG activity may be the result of EMG, which is not desirable for BCI applications.

=> We appreciate your valuable comments. From our dataset storage, we found 20 trials (per class) of real hand movement for each subject; such additional information would be added to GiGaDB (currently, link was closed. Thus, we asked link to be open for data update purpose.). According to reviewer's comments, we analyzed the EMG data and found that high frequency broad band activity (50-250 Hz) increased when subjects conducted the finger movements (shown in Figure 3). Considering this observation, we investigated whether the broad band power is correlated with labels of time points. We found that calculating the correlation between EEG and EMG is not easy. Thus, if EMG activity is correlated with ground truth information, we infer that the trial may be correlated to EEG. At first, we attempted this approach to real hand movement data and found that for most trials, significant correlation (corrected $p < 0.01$) between time labels and EMG activity was observed. Finally, we applied the same procedure to motor imagery data and figured out which trials were strongly correlated with EMG. Such EMG correlated trials were indexed and discarded for BCI classification analysis. To reflect reviewer's comments, we revised the manuscript as follows:

Section of 'Experiment and datasets'

"Real hand movement. Before beginning the motor imagery experiment, we asked subjects to conduct real hand movements. Subjects sat in a chair with armrests and watched a monitor. At the beginning of each trial, the monitor showed a black screen with a fixation cross for 2 seconds; the subject was then ready to perform hand movements (once the black screen gave a ready sign to the subject). As shown in Figure 2, one of two instructions ("left hand" or "right hand") appeared randomly on the screen for 3 seconds, and subjects were asked to move the appropriate hand depending on the instruction given. After the movement, when the blank screen reappeared, the subject was given a break for a random 4.1 to 4.8 seconds. These processes were repeated 20 times for one class (one run), and one run was performed.

MI experiment. The MI experiment was conducted with the same paradigm as the real hand movement experiment. Subjects were asked to imagine the hand movement depending on the instruction given. Five or six runs were performed during the MI experiment. After each run, we calculated the classification accuracy over one run and gave the subject feedback to increase motivation. Between each run, a maximum 4-minute break was given depending on the subject's demands."

Please see to Figure 4 in the revision.

Section of ‘Data format and structure’

“The MATLAB structure of the EEG (1st to 64th channel) and EMG (65th to 68th channel) data (‘*.mat’) for each subject is shown below:

- rest: resting state with eyes-open condition
- noise:
  – Eyeball movement up/down, 5 sec. x 2
  – Eyeball movement left/right, 5 sec. x 2
  – Jaw clenching, 5 sec. x 2
  – Head movement up/down, 5 sec. x 2
  – Head movement left/right, 5 sec. x 2
- imagery_left: 100 or 120 trials of left hand MI
- imagery_right: 100 or 120 trials of right hand MI
- n_imagery_trials: 100 or 120 trials for each MI class
- imagery_event: value “1” represents onset for each MI trial
- movement_left: 20 trials of real left hand movement
- movement_right: 20 trials of real right hand movement
- n_movement_trials: 20 trials for each real hand movement class
- movement_event: value “1” represents onset for each movement trial
- frame: temporal range of a trial in milliseconds
- srate: sampling rate
- senloc: 3D sensor locations
- psenloc: sensor location projected to unit sphere
- subject: subject’s two-digit ID - ‘s#’
- comment: comments for the subject
- bad_trial_indices
  – bad trials determined by voltage
  – bad trials correlated with EMG activity.”

Section of ‘Method’

“Secondly, we investigated whether each trial is correlated with EMG (e.g., real hand movement) adopting [13]’s idea which was using correlation between class labels and EMG activity. In the prescreening of EMG in the real hand movement experiment, we observed high frequency activity (50–250 Hz) during real hand movement. We calculated Spearman’s correlation (non-parametric)—which is less sensitive to outliers in the data—between the EMG power of high frequency activity and the label of time points, as follows:

1. High-pass filtering of all EMG trials above 0.5 Hz to remove drifts;
2. Common average reference;
3. Band-pass filtering of all trials with 50–250 Hz;
4. Hilbert transform;
5. Take absolute and squared magnitude for each complex value of all trials;
6. Extract data in the resting window (-1000–0 msec) and the task-related window (0–3000 msec) for each trial;
7. Prepare labels for each time points within a trial:
   – Tag ‘-1’ value for time points in the resting window;
   – Tag ‘+1’ value for time points in the task-related window;
8. Calculate Spearman’s correlation between squared magnitudes and label of time points
9. Permutation test over time points within a trial:
   – Calculated Spearman’s correlation between permuted features and labels;
   – Repeat 100 times;
   – Make probability density function (PDF) of the values of Spearman’s correlation;
   – Calculate p-values (one tailed test in the positive direction) over all trials and four EMG channels;

10. If false discovery rate (FDR) corrected p-value is smaller than 0.01, then it is a trial correlated with EMG.
Finally, the EMG correlated EEG trial indexes were added for each subject dataset, as shown in the section titled 'Data format and structure."

Section of 'Results'

"Percentage of bad trials. We calculated the percentage of bad trials for each subject, as shown in Figure 4A. The percentages of bad trials within the spectral and temporal discriminative ranges were below 5% for most subjects. Furthermore, we calculated the percentage of EMG trials correlated with labels of time points for each MI trial, as shown in Figure 4B. Two subjects (s29 and s34) showed more than 90% of their trials were correlated with EMG. Most of the trials demonstrated a greater power of high frequency EMG (50–250 Hz) in the task-related period after onset than the resting period before onset. Rest of subjects has at least 10 trials. The literature [8] showed that the upper confidence limits of chance with α = 5% were 70% (classification accuracy) in a 2-class problem with 10 trials for each class. If a subject has higher accuracy than the random chance level depending on the number of trials, we classified the subjects into discriminative group. On the other hand, we applied the same method to real hand movement trials to test our method. Most of the trials were correlated to the power of high frequency EMG. Here, although we set the p-value threshold as 0.05, a few trials were not correlated with the labels of resting or task-related states. Thus, our threshold was 0.01; we applied the same threshold to MI data.
Most existing studies detected EMG activity through manual monitoring. They recorded EMG and EEG simultaneously and monitored EMG burst during the experiment. On the other hand, in a literature [14], the resting state of EMG was recorded and the significant threshold from the resting EMG was defined. Furthermore, in another literature [13], correlation values between target position (cursor movement control application in BCI) and EMG activity were calculated, and they were compared with the correlation values of EMG-class labels and EEG-class labels. Also, according to the literature [14], t-values between the EMGs of the operant hand and the non-operant hand were calculated. We believed that the correlation between EMG activity and time point labels within a trial could provide the solution for detecting which EEG trial was correlated to EMG. We attempted the voltage thresholding method, but there were trials correlated with EMG even EMG activity was smaller than threshold. We also tried to compare the voltage distribution between the resting state and task-related EMG, but there were trials correlated to EMG although the distribution of EMG of a trial has a distribution similar to that of the resting state. Finally, Thirty-three subjects had higher classification accuracy than their own random chance (yellow diamond marker) with a confidence level of α = 5%, as shown in Figure 4C."

Q2) Figure 5A does not provide a compelling discrimination between left and right or across spatial regions. This should be presented in a more meaningful fashion. Moreover, the ERD activity from the topographies in figure 5B is curiously concentrated in the parietal and occipital areas, which should be characteristically observed over the motor cortex with more pronounced lateralization. This leads to concerns about whether the data is representative of the expected, well-established MI activity or is somehow corrupted. In summary, the data set is potentially useful to MI BCI researchers if further validated as suggested.

=> We appreciate reviewer's careful and good comments. With such comments, our manuscript was very improved, we believe. After more validation was attempted, we revised Figure 5 and the manuscript as follows:

See to Figure 5 in revised manuscript (new figure was added)

Section of 'Results'

"ERD/ERS. The ERD/ERS results of mu rhythm (8–14 Hz) are depicted in Figure 5. Figure 5A shows the grand averaged ERD/ERS (%) of C3 and C4 channel over the 33 subjects who has discriminative information (as shown in Figure 4C). The powers of mu rhythm in C3 and C4 channel decreased in both left and right

hand MI. Contralateral channel showed bigger desynchronization in corresponding class. The last row in Figure 5A shows the difference over time of ERD/ERS in C3 and C4 channels. The C4 channel showed a bigger difference than the C3 channel. Figure 5B shows the topographies at specific time points, for instance, 500, 1000, 1500, and 2000 msec. Those time points are marked in Figure 5A as a cyan colored vertical line. At 500 msec, the fronto-central and occipital areas were activated ('alpha inhibition [1,15]'). The activation of the occipital area may be related to processing of visual stimulus. The fronto-central area may be related to the frontal motor area for planning motor imagery [16,17]. At 1000 msec, contralateral channels showed bigger ERD than ipsilateral channels. For left hand MI, the right central and parietal areas showed bigger ERD than the left hemisphere. At 1500 msec, the frontal area was activated. We expect that the activation of the frontal area is related to control or modulation of other sensorimotor areas [16,17]. In Figure 5C, bar graphs of left hand MI show that the contralateral ERD (C4 channel) is stronger than the ipsilateral ERD (C3 channel)."

Reviewer #2

COMMENTS TO THE AUTHOR(S)
Review for „ GigaScience", Manuscript "EEG datasets for motor image brain computer interface" by Cho and colleagues.

Cho and colleagues present a dataset from 52 individuals. The dataset comprises electrophysiological (EEG) data during motor imagery (MI) and other, non-task related, tasks; 3D EEG electrode location as well as psychological and physiological questionnaires. With the provision of this, comparably large and rich dataset Cho and colleagues allow further investigation of a highly important issue in the field, the viability and reliability of MI-based brain-computer interface (BCI) and neurofeedback (NF) implementations.

Background and Purpose
Q1) "Thus, MI BCI is an almost ideal form of BCI."
There are many different BCI (application-centered approaches, e.g. steering a wheelchair) and NF (user-centered, e.g. modulation of cortical activation in the context of motor rehabilitation) implementations, whereby each of them has its advantages and disadvantages. Up to now the ideal BCI/NF does not exist and which of the different implementations is the most optimal one depends largely on the application and the user. Therefore, please rephrase the corresponding section.
The review by Grosse-Wentrup and Schlölkopf "A Review of Performance Variations in SMR-based Brain-Computer Interfaces (BCIs)" (2013) should not be missing in an introduction about inter- and intra-individual differences in MI BCI/NF performance.

=> We appreciate reviewer's valuable comments. To reflect reviewer's comments, we added additional references and revised the manuscript as follows:

Section of 'Background and Purpose'

"Motor imagery (MI) based brain computer interface (BCI) has attracted great interest recently. Compared with other BCI paradigms, MI BCI can provide users with direct communication without any limb movement or external stimulus (for example, P300 based BCI). MI BCI uses "induced" brain activity [1] from the cortex, rather than "evoked" brain activity. Although the concept of MI BCI is fascinating, it has many obstacles. Among these is the fact that BCI researchers have tended to focus on subject-to-subject transfer (training subject-independent algorithm). To achieve effective subject-to-subject transfer, it is important to understand the variations in performance between subjects [2]. Predicting a subject's performance by using the resting state, or background noise from EEG, are some examples of this [3–5]."

Experimental Design

Q2) Please specify if written or verbal consent was obtained from the participants.

=> We added more detailed information as follows:

Section of ‘Subjects’

“...All subjects gave written informed consent to collect information on brain signals and were paid for their participation. The data collected were used only for research purposes.”

Q3) Were EMG data recorded with the same system/ sampling rate as the EEG data? Please include information.

=> EMG was recorded with the same system and the same sample rate as EEG. We addressed this point as follows:

Section of ‘Recording software and device’

“... Furthermore, we simultaneously recorded EMG as well as EEG with the same system and sampling rate to check actual hand movements. Two EMG electrodes were attached to the flexor digitorum profundus and extensor digitorum on each arm.”

Q4) Please add reference for visual and kinesthetic MI (e.g. "Imagery of motor actions: Differential effects of kinesthetic and visual-motor mode imagery in single-trial EEG" by Neuper, N., Scherer, R., Reiner, M., Pfurtscheller, G. 2005)

=> We added this reference as follows:

Section of ‘Motor imagery instructions’

“ .... When imagining the movement, we asked subjects to imagine the kinesthetic experience [7], rather than imagining the visual experience.”

Q5) It seems that the link in the text to table 1 and table 2 is interchanged, please check.

=> We corrected this mistake.

Q6) In the section "Data format and structure" is mentioned: "Subject:; subject two digit ID - 's##'", whereby in the section "Subjects" is written "... ID was denoted and indexed as s1, s2, ..., s52". Please align the two statements.

=> We corrected this mistake.

Reliability
Q7) Please provide information about filter type and order.

=> We added details on filters as follows:

Section of ‘Method’

“For preprocessing, we used Butterworth filtering with 4th order for high-pass and band-pass filtering. …”

Q8) "The percentage of bad trials was estimated for each subject." Why estimated and not exactly calculated?

=> We used ‘estimated’ since the number of total trials were not the same for all subjects. However, to reflect reviewer’s comment, we corrected it. Further, we added all bad trial indices into the dataset.

Q9) Was classification based on left versus right hand MI or on left and/or right hand MI versus baseline? Please clarify.

=> To clarify such point, we added more detailed information as follow:

Section of ‘Method’

“Lastly, we validated the discriminability of the left versus right hand MI EEG data as classification accuracy. …”

Results
Q10) "…, as shown in Figure 4" → "…, as shown in Figure 4A"

=> We corrected this mistake.

Q11) Please add x- and y-labels for Fig. 4A

=> We added labels in Figure 4. Please see to Figure 4 in the revision.

Q12) It would be more informative to depict a larger time window in Figure 5A, i.e. from -1 s to 4 s in order to show in addition to the MI-related ERD also the rebound, or ERS, following MI. Why was this time window (500-700 ms) chosen for the topographical illustration? Such a short time window is rather untypical for MI tasks lasting several seconds. Instead, or in addition, the topography of the entire MI time interval (minus some time at the beginning and end, e.g. 500-2500 ms) should be illustrated. This might also lead to a shift in the activity pattern, from the visually dominant pattern illustrated in Figure 5B, towards a more central (sensorimotor) pattern. Why are the scales in Figure 5A, 5B and 5C not identical (ERD(%) versus -log(ERD))? The same scale would allow better comparability.

=> To reflect reviewer’s valuable comments, we updated Figure 5 and revised ERD/ERS section as follows:

Please see to Figure 5 in the revision.

Section of ‘Results’

“

ERD/ERS. The ERD/ERS results of mu rhythm (8–14 Hz) are depicted in Figure 5. Figure 5A shows the grand averaged ERD/ERS (%) of C3 and C4 channel over the 33 subjects who has discriminative information (as shown in Figure 4C). The powers of mu rhythm in C3 and C4 channel decreased in both left and right hand MI. Contralateral channel showed bigger desynchronization in corresponding class. The last row in Figure 5A shows the difference over time of ERD/ERS in C3 and C4 channels. The C4 channel showed a bigger

difference than the C3 channel. Figure 5B shows the topographies at specific time points, for instance, 500, 1000, 1500, and 2000 msec. Those time points are marked in Figure 5A as a cyan colored vertical line. At 500 msec, the fronto-central and occipital areas were activated (‘alpha inhibition [1,15]’). The activation of the occipital area may be related to processing of visual stimulus. The fronto-central area may be related to the frontal motor area for planning motor imagery [16,17]. At 1000 msec, contralateral channels showed bigger ERD than ipsilateral channels. For left hand MI, the right central and parietal areas showed bigger ERD than the left hemisphere. At 1500 msec, the frontal area was activated. We expect that the activation of the frontal area is related to control or modulation of other sensorimotor areas [16,17]. In Figure 5C, bar graphs of left hand MI show that the contralateral ERD (C4 channel) is stronger than the ipsilateral ERD (C3 channel).”
