# Supplementary material for: EEG datasets for motor imagery brain–computer interface
Source: Gigascience. 2017 May 4;6(7):1–8. doi: 10.1093/gigascience/gix034 (PMC5493744; doi:10.1093/gigascience/gix034)
Supplement: Response_to_reviewer_comments_revision_1.pdf [file gix034_Response_to_reviewer_comments_revision_1.pdf]

Comment 1. The manuscript has been significantly improved in response to the reviewers' comments. However, there are still some concerns. The percentage of bad trials (correlated with EMG) shown in Figure 4 is quite high and limits the utility of a significant portion of the data.

=> We appreciate your valuable comment. Actually, we applied significant p-value of 0.01 for determining bad trials correlated with EMG. However, we observed that a number of trials satisfied significant p-value even though correlation values were quite small, which means that our criterion seems not good. To avoid these cases, we considered correlation threshold as an additional criterion. We applied the same method to real hand movement trials and observed that most trials were strongly correlated to the power of high frequency EMG (50-250Hz) and their correlation values (more than 85%) were higher than 0.8. Thus, a reasonable correlation threshold was empirically determined as 0.8. Finally, when EMG signal in a motor imagery trial has greater correlation than this threshold (0.8) and the p-value is small than 0.01 (Bonferroni corrected), then the trial was classified to a bad trial. With this updated approach, we reduced bad trials notably; thus, 38 subjects were classified to reasonably discriminative group (33 subjects in the previous revised manuscript). Corresponding sentences, paragraphs, figures, and figure captions were revised and updated accordingly as follows:

Figure 4 : it was updated accordingly.

Methods in the ‘Reliability’ Section
In 2nd item

“10. If Bonferroni corrected p-value is smaller than 0.01 and the correlation value is greater than 0.8, then it is declared as a bad trial correlated with EMG.”

Results Section

“Percentage of bad trials. “We calculated the percentage of bad trials for each subject, as shown in Figure 4A. The percentages of bad trials within the spectral and temporal discriminative ranges were below 5% for most subjects. Furthermore, we calculated the percentage of EMG trials correlated with labels of time points for each MI trial, as shown in Figure 4B. Two subjects (s29 and s34) showed that more than 90% of their trials were correlated with EMG; most of the trials demonstrated a greater power of high frequency EMG (50–250 Hz) in the task-related period after onset than the resting period before onset. Thus, these two subjects were declared as bad subjects and were discarded in the further analysis. Rest of subjects has at least 10 trials per class. The literature [8] showed that the upper confidence limits of chance with α = 5% were 70% (classification accuracy) in a 2-class problem with 10 trials for each class. If a subject has higher accuracy than the random chance level depending on the number of trials [8], we classified the subjects into discriminative group. On the other hand, we applied the same method to real hand movement trials to test our method. We observed that most trials (more than 85%) were correlated to the power of high frequency EMG (50-250Hz) and the correlation values were higher than 0.8. Here, although we set the p-value threshold as 0.05, a few trials were not correlated with the labels of resting or task-related states. Thus, our threshold of p-value was set to 0.01. Furthermore, according to the observed correlation distributions of real hand movement data, we set 0.8 as a correlation threshold. Finally, if the correlation value is greater than 0.8 threshold and the p-value is smaller than 0.01 in MI datasets, the trial was classified to bad trial correlated with real hand movement.”

Comment 2. For Figure 5, the revised "bad trial" exclusion criteria did not appreciably improve the foci of the activity over the sensorimotor cortex and there are still larger than expected changes over the occipital regions. It is suggested to apply a Large Laplacian spatial filter to see if the expected topographies (prominent focal changes over the left and right hand areas of SMC and minimal changes elsewhere) can be obtained. It may be beneficial to investigate the topographies for each subject individually to see if the activity is consistent across subjects or if several subjects are exhibiting a non-characteristic response.

=> We appreciated your valuable comment. According to your suggestion, Laplacian filtering was applied to our data. We observed the prominent focal changes in SMC, however, some alpha ERDs in occipital still remained. With simple detection algorithm, most of trials were found to have occipital alpha ERD, which may be related to processing for visual instruction of left/right motor imagery. This is quite relevant to findings in the literature [Figure 8a in Pfurtscheller et al 1999 Clinical Neurophysiology, Figure 1 in Neuper et al 2005 Cognitive brain research, Figure 2 in McFarland et al 2000 Brain Topography]. We noted that individual subjects showed similarly consistent activations in both SMC and occipital.

More importantly, it should be checked whether the occipital alpha ERD is discriminative between left/right motor imagery movements. CSP filters from 38 subject's left and right MI covariance matrices were generated, as shown in Figure 6. It was observed that prominent CSP weights exist around SMC only, but not in occipital; thus, these occipital alpha ERD were not influenced on the quality of our MI data. In this revision, these CSP filters were added in Figure 6 and corresponding sentences were revised as follow:

Figure 6 : it was updated by adding illustration of two CSP filters

ERD/ERS in the 'Results' Section

"At 500 msec, the occipital areas were activated ('alpha inhibition [1,15]'). The occipital alpha ERD was continued to 2000 msec. We also tested whether each trial contains occipital alpha ERD or not by using permutation test, just like EMG trial detection. We found most of trials have the occipital alpha ERD for all subjects. Common spatial pattern (CSP) filters (representing the filtering weight for each channel) trained by the 38 subjects were estimated, as shown in Figure 6A; prominent CSP weights existed around SMC only, but not in occipital. It means that the occipital alpha ERD may not affect the class labeled data. It is expected that the activation of the occipital area may be related to processing of visual stimulus. However, the occipital alpha activity was not influenced on the quality of our left/right motor imagery data."
