# Supplementary material for: EEG datasets for motor imagery brain–computer interface
Source: Gigascience. 2017 May 4;6(7):1–8. doi: 10.1093/gigascience/gix034 (PMC5493744; doi:10.1093/gigascience/gix034)
Supplement: Response_to_reviewer_comments_revision_2.pdf [file gix034_Response_to_reviewer_comments_revision_2.pdf]

We have done all, as you requested.
