# Supplementary material for: EEG datasets for motor imagery brain–computer interface
Source: Gigascience. 2017 May 4;6(7):1–8. doi: 10.1093/gigascience/gix034 (PMC5493744; doi:10.1093/gigascience/gix034)
Supplement: Reviewer_1_Report_(Original_Submission).pdf [file gix034_Reviewer_1_Report_(Original_Submission).pdf]

Reviewer Report

Title: "EEG datasets for motor imagery brain computer interface"

Version: Original Submission **Date:** 11/16/2016

Reviewer name: Dean Krusienski

Reviewer Comments to Author:

This manuscript describes an EEG motor imagery (MI) data set collected from 52 subjects. The analysis is primarily to confirm the validity of the data and is not novel. Such consistently-collected and comparatively large data sets are of interest to EEG-based brain-computer interface (BCI) researchers. Another similar and large EEG MI data set is available on PhysioNet, but additional large and consistent data sets are beneficial to the research community. The present data set is also unique in that it includes other non-EEG measurements, such as EMG and EOG, and subjective evaluations that can be useful for a variety of analyses. There are a few items that should be addressed in order to improve the utility of this data set. For MI data, it is desirable to have EEG from actual movements as a reference of the spatio-spectral characteristics of the EEG during movement for each subject. Without this, it is difficult to determine if the MI non-responders weren't performing the imagery properly, do not have prominent sensorimotor rhythms, etc. Since EMG and other non-EEG measurements have been acquired, for the data validation, it is desirable to validate or screen for trials containing EMG that was correlated to the MI. Otherwise, some part of the EEG activity may be the result of EMG, which is not desirable for BCI applications. Figure 5A does not provide a compelling discrimination between left and right or across spatial regions. This should be presented in a more meaningful fashion. Moreover, the ERD activity from the topographies in figure 5B is curiously concentrated in the parietal and occipital areas, which should be characteristically observed over the motor cortex with more pronounced lateralization. This leads to concerns about whether the data is representative of the expected, well-established MI activity or is somehow corrupted. In summary, the data set is potentially useful to MI BCI researchers if further validated as suggested.

Level of Interest

Please indicate how interesting you found the manuscript: An article whose findings are important to those with closely related research interests

Quality of Written English

Please indicate the quality of language in the manuscript: Acceptable

Declaration of Competing Interests

Please complete a declaration of competing interests, considering the following questions:

- Have you in the past five years received reimbursements, fees, funding, or salary from an organisation that may in any way gain or lose financially from the publication of this manuscript, either now or in the future?
- Do you hold any stocks or shares in an organisation that may in any way gain or lose financially from the publication of this manuscript, either now or in the future?

- Do you hold or are you currently applying for any patents relating to the content of the manuscript?
- Have you received reimbursements, fees, funding, or salary from an organization that holds or has applied for patents relating to the content of the manuscript?
- Do you have any other financial competing interests?
- Do you have any non-financial competing interests in relation to this paper?

If you can answer no to all of the above, write 'I declare that I have no competing interests' below. If your reply is yes to any, please give details below.

I declare that I have no competing interests

I agree to the open peer review policy of the journal. I understand that my name will be included on my report to the authors and, if the manuscript is accepted for publication, my named report including any attachments I upload will be posted on the website along with the authors' responses. I agree for my report to be made available under an Open Access Creative Commons CC-BY license (http://creativecommons.org/licenses/by/4.0/). I understand that any comments which I do not wish to be included in my named report can be included as confidential comments to the editors, which will not be published.

I agree to the open peer review policy of the journal
