# Supplementary material for: EEG datasets for motor imagery brain–computer interface
Source: Gigascience. 2017 May 4;6(7):1–8. doi: 10.1093/gigascience/gix034 (PMC5493744; doi:10.1093/gigascience/gix034)
Supplement: Reviewer_1_Report_(revision_1).pdf [file gix034_Reviewer_1_Report_(revision_1).pdf]

Reviewer Report

Title: "EEG datasets for motor imagery brain computer interface"

Version: Revision 1 **Date:** 1/16/2017

Reviewer name: Dean Krusienski

Reviewer Comments to Author:

The manuscript has been significantly improved in response to the reviewers' comments. However, there are still some concerns. The percentage of bad trials (correlated with EMG) shown in Figure 4 is quite high and limits the utility of a significant portion of the data. For Figure 5, the revised "bad trial" exclusion criteria did not appreciably improve the foci of the activity over the sensorimotor cortex and there are still larger than expected changes over the occipital regions. It is suggested to apply a Large Laplacian spatial filter to see if the expected topographies (prominent focal changes over the left and right hand areas of SMC and minimal changes elsewhere) can be obtained. It may be beneficial to investigate the topographies for each subject individually to see if the activity is consistent across subjects or if several subjects are exhibiting a non-characteristic response.

Level of Interest

Please indicate how interesting you found the manuscript: An article whose findings are important to those with closely related research interests

Quality of Written English

Please indicate the quality of language in the manuscript: Acceptable

Declaration of Competing Interests

Please complete a declaration of competing interests, considering the following questions:

- Have you in the past five years received reimbursements, fees, funding, or salary from an organisation that may in any way gain or lose financially from the publication of this manuscript, either now or in the future?
- Do you hold any stocks or shares in an organisation that may in any way gain or lose financially from the publication of this manuscript, either now or in the future?
- Do you hold or are you currently applying for any patents relating to the content of the manuscript?
- Have you received reimbursements, fees, funding, or salary from an organization that holds or has applied for patents relating to the content of the manuscript?
- Do you have any other financial competing interests?
- Do you have any non-financial competing interests in relation to this paper?

If you can answer no to all of the above, write 'I declare that I have no competing interests' below. If your reply is yes to any, please give details below.

I declare that I have no competing interests

I agree to the open peer review policy of the journal. I understand that my name will be included on my report to the authors and, if the manuscript is accepted for publication, my named report including any attachments I upload will be posted on the website along with the authors' responses. I agree for my report to be made available under an Open Access Creative Commons CC-BY license (http://creativecommons.org/licenses/by/4.0/). I understand that any comments which I do not wish to be included in my named report can be included as confidential comments to the editors, which will not be published.

I agree to the open peer review policy of the journal
