# Supplementary material for: EEG datasets for motor imagery brain–computer interface
Source: Gigascience. 2017 May 4;6(7):1–8. doi: 10.1093/gigascience/gix034 (PMC5493744; doi:10.1093/gigascience/gix034)
Supplement: Reviewer_2_Report_(Original_Submission).pdf [file gix034_Reviewer_2_Report_(Original_Submission).pdf]

Reviewer Report

Title: "EEG datasets for motor imagery brain computer interface"

Version: Original **Date:** 12/12/2016

Reviewer name: Catharina Zich

Reviewer Comments to Author:

Review for „ GigaScience", Manuscript "EEG datasets for motor imagerz brain computer interface" by Cho and colleagues.Cho and colleagues present a dataset from 52 individuals. The dataset comprises electrophysiological (EEG) data during motor imagery (MI) and other, non-task related, tasks; 3D EEG electrode location as well as psychological and physiological questionnaires. With the provision of this, comparably large and rich dataset Cho and colleagues allow further investigation of a highly important issue in the field, the viability and reliability of MI-based brain-computer interface (BCI) and neurofeedback (NF) implementations. Background and Purpose* "Thus, MI BCI is an almost ideal form of BCI." There are many different BCI (application-centered approaches, e.g. steering a wheelchair) and NF (user-centered, e.g. modulation of cortical activation in the context of motor rehabilitation) implementations, whereby each of them has its advantages and disadvantages. Up to now the ideal BCI/NF does not exist and which of the different implementations is the most optimal one depends largely on the application and the user. Therefore, please rephrase the corresponding section. * The review by Grosse-Wentrup and Schlölkopf "A Review of Performance Variations in SMR-based Brain-Computer Interfaces (BCIs)" (2013) should not be missing in an introduction about inter- and intra-individual differences in MI BCI/NF performance. Experimental Design* Please specify if written or verbal consent was obtained from the participants. * Were EMG data recorded with the same system/ sampling rate as the EEG data? Please include information. * Please add reference for visual and kinesthetic MI (e.g. "Imagery of motor actions: Differential effects of kinesthetic and visual-motor mode imagery in single-trial EEG" by Neuper, N., Scherer, R., Reiner, M., Pfurtscheller, G. 2005) * It seems that the link in the text to table 1 and table 2 is interchanged, please check. * In the section "Data format and structure" is mentioned: "Subject:; subject two digit ID - 's##'", whereby in the section "Subjects" is written "... ID was denoted and indexed as s1, s2, ..., s52". Please align the two statements.Reliability* Please provide information about filter type and order.*"The percentage of bad trials was estimated for each subject." Why estimated and not exactly calculated?* Was classification based on left versus right hand MI or on left and/or right hand MI versus baseline? Please clarify.Results* "..., as shown in Figure 4" → "..., as shown in Figure 4A"* Please add x- and y-labels for Fig. A4* It would be more informative to depict a larger time window in figure 5A, i.e. from -1 s to 4 s in order to show in addition to the MI-related ERD also the rebound, or ERS, following MI. * Why was this time window (500-700 ms) chosen for the topographical illustration? Such a short time window is rather untypical for MI tasks lasting several seconds. Instead, or in addition, the topography of the entire MI time interval (minus some time at the beginning and end, e.g. 500-2500 ms) should be illustrated. This might also lead to a shift in the activity pattern, from the visually dominant pattern illustrated in Figure 5B, towards a more central (sensorimotor) pattern.* Why are the scales in Figure 5A, 5B and 5C not identical (ERD(%) versus -log(ERD))? The same scale would allow better comparability.

Level of Interest

Please indicate how interesting you found the manuscript: An article of importance in its field

Quality of Written English

Please indicate the quality of language in the manuscript: Acceptable

Declaration of Competing Interests

Please complete a declaration of competing interests, considering the following questions:

- Have you in the past five years received reimbursements, fees, funding, or salary from an organisation that may in any way gain or lose financially from the publication of this manuscript, either now or in the future?
- Do you hold any stocks or shares in an organisation that may in any way gain or lose financially from the publication of this manuscript, either now or in the future?
- Do you hold or are you currently applying for any patents relating to the content of the manuscript?
- Have you received reimbursements, fees, funding, or salary from an organization that holds or has applied for patents relating to the content of the manuscript?
- Do you have any other financial competing interests?
- Do you have any non-financial competing interests in relation to this paper?

If you can answer no to all of the above, write 'I declare that I have no competing interests' below. If your reply is yes to any, please give details below.

I declare that I have no competing interests

I agree to the open peer review policy of the journal. I understand that my name will be included on my report to the authors and, if the manuscript is accepted for publication, my named report including any attachments I upload will be posted on the website along with the authors' responses. I agree for my report to be made available under an Open Access Creative Commons CC-BY license (http://creativecommons.org/licenses/by/4.0/). I understand that any comments which I do not wish to be included in my named report can be included as confidential comments to the editors, which will not be published.

I agree to the open peer review policy of the journal
